# Bacterial Cytochrome P450 Involvement in the Biodegradation of Fluorinated Pyrethroids

**DOI:** 10.3390/jox15020058

**Published:** 2025-04-18

**Authors:** Mohd Faheem Khan, Jun Liao, Zhenyang Liu, Gaurav Chugh

**Affiliations:** 1 School of Agriculture and Food Science, University College Dublin, Belfield, Dublin 4, D04 V1W8 Dublin, Ireland; 2 China Guangzhou Dublin International College of Life Sciences & Technology, South China Agricultural University, 483 Wushan Road, Tianhe District, Guangzhou 510642, China; 3 School of Biomolecular and Biomedical Science, University College Dublin, Belfield, Dublin 4, D04 V1W8 Dublin, Ireland

**Keywords:** pesticides, cyfluthrin, cyhalothrin, soil bacteria, biodegradation, cytochrome P450

## Abstract

Fluorinated pyrethroids, such as cyfluthrin and cyhalothrin, are more effective insecticides due to their enhanced stability and lipophilicity. However, they pose greater risks to non-target organisms. Their persistence in the environment and accumulation in tissues can lead to increased toxicity and ecological concerns. This study investigates the biodegradation of the fluorinated pyrethroids β-cyfluthrin (BCF) and λ-cyhalothrin (LCH) using a newly isolated *Bacillus* sp. MFK14 from a garden soil microbial consortium. Initial screening using ^19^F NMR analysis showed that the microbial consortium degraded both pyrethroids, leading to the isolation of *Bacillus* sp. MFK14. Subsequent GC-MS analysis revealed various degradation intermediates in both pyrethroids after incubation with *Bacillus* sp. MFK14. Notably, *Bacillus* sp. MFK14 completely degraded β-cyfluthrin and λ-cyhalothrin within 48 h at 30 °C. Fluoride ions from β-cyfluthrin and trifluoroacetic acid (TFA) from λ-cyhalothrin were detected as the end-products by ^19^F NMR analysis of the aqueous fraction. The pathway of the degradation was proposed for both the pyrethroids indicating shared biodegradation pathways despite different fluorinations. Inhibition studies with 1-ABT suggested the involvement of bacterial cytochrome P450 (CYP) enzymes in their biodegradation. The CYPome of *Bacillus* sp. MFK14 includes 23 CYP variants that showed significant sequence similarity to known bacterial CYPs, suggesting potential roles in pyrethroid biodegradation and environmental persistence. These findings highlight the potential for bioremediation of fluorinated pesticides, offering an environmentally sustainable approach to mitigate their ecological impact.

## 1. Introduction

Excessive pesticide use in agriculture adversely impacts insects, wildlife, aquatic life, plants, and human health. Among these pesticides, pyrethroids are a potent class of synthetic insecticides that effectively target a wide array of agricultural pests, mosquitoes, and flies by disrupting their nervous systems, leading to paralysis and death [1]. Compared to other insecticides, pyrethroids exhibit relatively mild toxicity to humans and mammals. However, fluorination of pyrethroids enhances their properties by increasing lipophilicity, stability, and binding affinity to biological targets, which improves their insecticidal efficacy but also heightens toxicity to non-target organisms, including humans and animals [2]. Notably, fluorinated pyrethroids—such as transfluthrin, tefluthrin, flumethrin, cyhalothrin, cyfluthrin, bifenthrin, and fluvalinate penetrate membranes more effectively, persist longer in the environment, and accumulate in biological tissues, leading to increased neurotoxicity, metabolic resistance, and potential endocrine disruption [2]. For instance, Martínez et al. investigated cyfluthrin-induced neurotoxicity in humans, revealing that it led to significant increases in ROS, lipid peroxides, and nitric oxide, along with altered gene expression related to apoptosis and inflammation due to induced oxidative stress [3]. Numerous other studies have reported their high toxicity to aquatic organisms and the adverse effects of long-term exposure on human semen quality [4,5]. Due to their environmental persistence, pyrethroids are widely detected in soil, water, and air, impacting various ecosystems and raising significant health and environmental concerns.

Microbial biodegradation has emerged as a potential strategy for mitigating pyrethroid contamination, particularly given the resistance of these compounds to metabolic detoxification pathways. Various studies have demonstrated that bacteria such as *Pseudomonas aeruginosa*, *Bacillus licheniformis*, and *Bacillus subtilis*, as well as fungi like *Aspergillus niger*, *Cunninghamella elegans*, and *Fusarium proliferatum*, can effectively degrade pyrethroid residues in water and soil [6]. For instance, Li et al. reported that a novel soil bacterial consortium effectively degraded β-cyfluthrin, with different bacterial genera like *Enterobacter*, *Microbacterium*, *Ochrobactrum*, and *Pseudomonas* playing key roles at successive stages of the degradation process [7]. In bacteria such as *Bacillus subtilis* BSF01, *Bacillus licheniformis* B-1, and *Aspergillus niger* YAT, pyrethroids are initially hydrolysed by carboxylesterase or pyrethroid hydrolase, which cleaves the main ester bond linking the cyclopropane and aromatic moieties [8,9]. Similarly, in fungi like *Cunninghamella elegans*, cytochrome P450 enzymes (CYP5208A3) mediate cleavage of the central ester bonds [10,11].

Cytochrome P450 enzymes (CYP) play a crucial role in the biodegradation of various environmental pollutants by catalysing oxidation reactions that enhance the solubility and breakdown of complex organic compounds, including pesticides, hydrocarbons, and pharmaceuticals [12]. While the role of fungal cytochrome P450 enzymes in pyrethroid degradation is well established, there are limited reports on bacterial cytochrome P450 enzymes in this process. Li et al. highlighted the significance of these enzymes in the late-stage metabolism of β-cyfluthrin, particularly in the breakdown of benzoate intermediates. However, the involvement of bacterial cytochrome P450 enzymes in the initial cleavage of pyrethroid ester bonds remains largely unreported [7]. Further degradation generally involves oxidation and cleavage of pyrethroid aromatic rings facilitated by enzymes such as phenol hydrolases, monooxygenases, and dioxygenases [13].

In addition, fluorinated xenobiotic compounds, including pesticides, pose a significant challenge to biodegradation due to the stability of the carbon–fluorine (C-F) bond—the strongest in organic chemistry. Microbial enzymes have not naturally evolved to efficiently cleave C-F bonds, making these compounds persist in the environment and complicating their microbial degradation [12].

In this study, we explore the biodegradation of the fluorinated pyrethroids β-cyfluthrin and λ-cyhalothrin using microbial isolates from garden soil. We identify bacterial strains capable of metabolising these compounds and examine the degradation pathways involved. The identification of degradation intermediates, including fluoride ions and trifluoroacetic acid, was achieved using GC-MS and ^19^F NMR analysis. Furthermore, inhibition studies confirmed the involvement of cytochrome P450 enzymes in multiple steps of the biodegradation process. These findings highlight the potential for an environmentally sustainable approach to mitigate the ecological impact of fluorinated pesticides. By elucidating the microbial degradation pathways and identifying key enzymatic players, this study paves the way for developing bioremediation strategies that harness naturally occurring bacteria to detoxify contaminated environments. Such approaches could reduce pesticide accumulation in soil and water, minimising harmful effects on ecosystems while promoting sustainable agricultural and environmental management practices.

## 2. Materials and Methods

### 2.1. Chemicals and Reagents

The pyrethroid insecticides β-cyfluthrin (PESTANAL^®^) and λ-cyhalothrin (PESTANAL^®^) were purchased from Sigma-Aldrich (Wicklow, Ireland). Other reagents and chemicals, including N-methyl-N-(trimethylsilyl)trifluoroacetamide (MSTFA), 1-aminobenzotriazole (1-ABT), phenylmethylsulfonyl fluoride (PMSF), p-nitrophenyl acetate (p-NPA), deuterium oxide (D_2_O), and methanol-d_4_, were also acquired from Sigma-Aldrich (Ireland).

### 2.2. Soil Microbe Isolation and Cultivation

Details on soil sample collection and cultivation of the soil microbial consortium were previously described by Khan et al. [14]. The microbial consortium isolated from the garden soil sample was cultured in 20 mL of tryptone soya broth (TSB) in a 100 mL Erlenmeyer flask at 30 °C with shaking at 200 rpm for 48 h. The inoculum was diluted up to 10^−6^, spread on tryptone soya agar (TSA) plates, and incubated overnight at 30 °C. Different colonies were grown overnight on M9 minimal media supplemented with pyrethroids as the sole carbon source (2 mg/mL), and a potential bacterial strain was selected based on growth, which was estimated by measuring optical density (OD_600_) using a Jenway 7315 spectrophotometer [15].

### 2.3. Whole Genome Sequencing and Phylogenetic Analysis

The isolated bacterial strain, capable of growing on pyrethroid as the sole carbon source, was named “MFK14” and streaked on a fresh TSA plate. A single colony was selected and sent for whole genome Illumina sequencing to MicrobesNG (Birmingham, UK). The genome sequence was then submitted to NCBI GenBank (Accession number is JBLMDV000000000). To find the 16S rRNA gene sequence, the genome sequence (in fasta format) was uploaded in NCBI BLASTn and chosen “16S ribosomal RNA sequences (Bacteria and Archaea)” from the Database menu. The homologous 16S rRNA sequences of the known bacteria were obtained using BLASTn for phylogenetic analysis. The Clustal Omega multiple sequence alignment program (https://www.ebi.ac.uk/jdispatcher/msa/clustalo; accessed on 3 January 2025) was used to align all the 16S rRNA gene sequences for the generation of a phylogenetic tree using iTOL v6 (https://itol.embl.de/).

### 2.4. Biodegradation of Pyrethroids and Metabolite Extraction

The isolated bacterial strain, now identified as *Bacillus* sp. MFK14, was grown in 20 mL TSB for 48 h at 30 °C with shaking at 200 rpm. The cultures were transferred into 50 mL Falcon tubes, and the cells were collected by centrifugation at 4000 rpm for 5 min. The cells were then washed and resuspended in 20 mL phosphate-buffered saline (PBS) solution (pH 7.4). The pesticides (2 mg) were incubated with the bacterial suspension at 30 °C with shaking at 200 rpm for 48 h. The control experiment was carried out without the addition of pesticides.

The supernatants were transferred to fresh Falcon tubes, and the cells were removed by centrifugation at 4000 rpm for 5 min. Organic-soluble metabolites were extracted from the supernatants by adding 20 mL of ethyl acetate, shaking the tubes for 5 min, and centrifuging at 4000 rpm for 2 min to separate the organic and aqueous layers. The top organic layer was collected into fresh Falcon tubes, and the solvent evaporated under a stream of N_2_ gas until approximately 1 mL of the sample remained. The samples were then transferred to 2 mL glass vials and further concentrated under N_2_ gas.

### 2.5. ^19^F NMR and GC-MS Analysis

The ^19^F nuclear magnetic resonance spectroscopy (^19^F NMR) spectra were acquired using a Varian 400 MHz spectrometer, and the sample preparation method for organic-soluble and aqueous metabolites was described previously by Khan and Murphy [15]. For the organic metabolites, the residue left after evaporation of extract in glass vials was redissolved in 800 µL of methanol-d_4_ and transferred to NMR tubes. For the aqueous metabolites, culture supernatants were freeze-dried, and the resulting residue was redissolved in 800 µL of D_2_O and transferred into NMR tubes for ^19^F NMR analysis.

The gas chromatography-mass spectrometry (GC–MS) was performed with a 7890B Agilent GC equipped with an HP-5MS column, and a 5977A mass-selective detector was used to identify organic biodegradation intermediates. The dried organic extracts were silylated by adding 20 µL MSTFA and heating at 100 °C for 30–60 min; the sample volume was adjusted to 0.5 mL with ethyl acetate prior to injection onto the HP-5MS capillary column (30 m × 0.25 mm × 0.33 μm). The sample injector was set to splitless mode and injected 1 µL onto the column, with the oven temperature held at 80 °C for 3 min and then increased to 300 °C at 10 °C/min, while the mass spectrometer operated in scan mode [16].

### 2.6. Inhibition Assays and CYPome Prediction

*Bacillus* sp. MFK14 cultures were pre-grown for 48 h, the growth media was replaced with PBS, and 1 mM 1-ABT was added. The cultures were then incubated at 30 °C, 200 rpm shaking for 1 h before the addition of fluorinated pyrethroids, and the incubation continued for 48 h. The supernatants were extracted, silylated, and analysed by GC–MS as previously described.

To study the primary ester bond hydrolysis of pyrethroids, the role of esterases or CYPs in the cell-free lysate of *Bacillus* sp. MFK14 (1 mL) was assessed using a colourimetric assay with p-NPA as the substrate. The cleavage of the ester bond released p-nitrophenol (p-NP), resulting in the development of a yellow colour. To evaluate the involvement of these enzymes, 1 mM 1-ABT was used to inhibit CYP activity, while 1 mM PMSF was used to inhibit esterase activity. If both enzymes are involved, their activity would be inhibited by the respective inhibitors when tested with the cell-free lysate (sonicated at 35% amplitude, 5 s on/5 s off for 10 min).

The sequences of bacterial cytochrome P450 (CYP) enzymes were collected from UniProtKB (https://www.uniprot.org/uniprotkb) and the Cytochrome P450 Homepage available on the University of Tennessee Health Science Center website (https://drnelson.uthsc.edu/bacteria/). Each sequence of the collected CYP was searched for CYP orthologs in the uncharacterised genome of *Bacillus* sp. MFK14 using NCBI tBLASTn. The percentage protein identity of CYP orthologs in *Bacillus* sp. MFK14 was then determined.

## 3. Results

### 3.1. Pyrethroid Biodegradation by Microbial Consortium Isolated from Garden Soil

**Figure 1:**
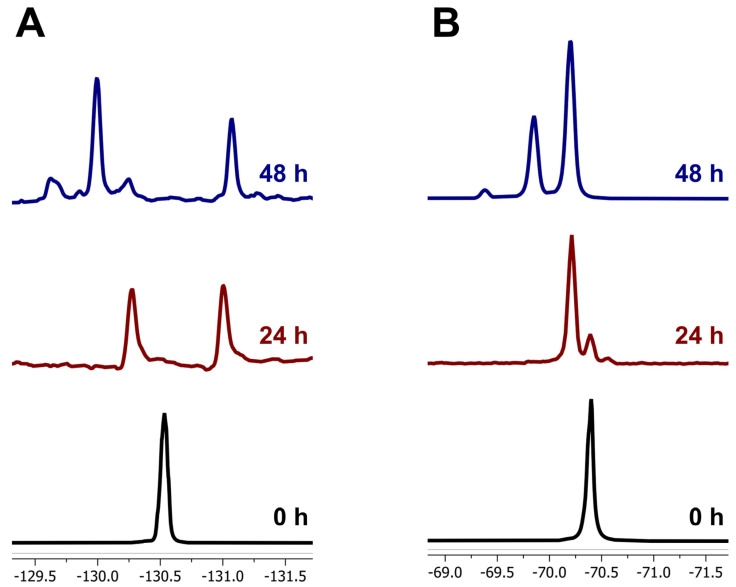
^19^F NMR analysis of biodegradation of fluorinated pyrethroid (**A**) β-cyfluthrin, and (**B**) λ-cyhalothrin by garden soil microbial consortium at different time incubations.

A consortium of garden soil microbes was screened for degradation of the fluorinated pyrethroids β-cyfluthrin and λ-cyhalothrin by enrichment. For β-cyfluthrin, the ^19^F NMR spectra showed the disappearance of the characteristic resonance at δ = −130.5 ppm, corresponding to the C-F bond on the aromatic moiety in the 0 h sample, and the appearance of two new resonances at δ = −130.2 and −131.0 ppm after 24 h of incubation with the soil microbial consortium. After 48 h, the resonance at δ = −131.0 ppm persisted, while the resonance at δ = −130.2 ppm significantly decreased, with the emergence of a major resonance at δ = −129.9 ppm and a minor resonance at δ = −129.6 ppm, indicating the formation of different fluorinated compounds due to microbial degradation (Figure 1A).

Similarly, for λ-cyhalothrin, the ^19^F NMR spectra showed the disappearance of the characteristic resonance at δ = −70.4 ppm, corresponding to the -CF_3_ group in the 0 h sample, and the emergence of a new resonance at δ = −70.1 ppm after 24 h, followed by the appearance of two additional resonances at δ = −69.8 and −69.4 ppm after 48 h, further indicating the microbial degradation of the substrate (Figure 1B).

### 3.2. Isolation and Identification of Bacterium Utilising Fluorinated Pyrethroids as a Carbon Source

**Figure 2:**
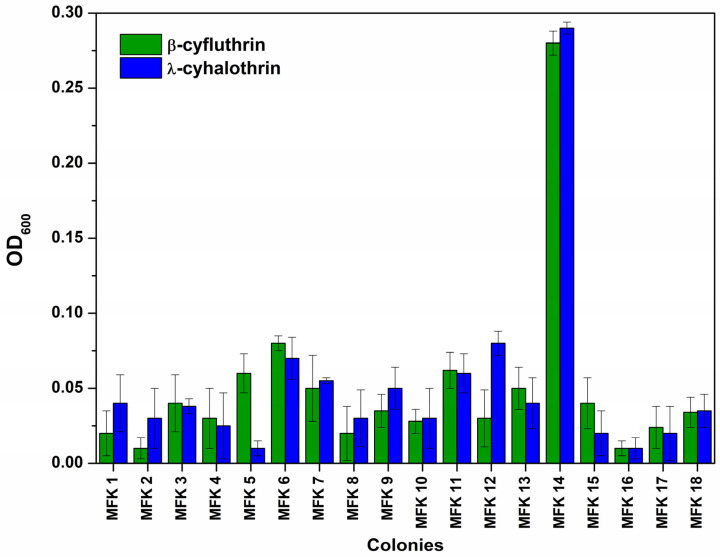
Screening of colonies grown on M9 minimal media supplemented with a pyrethroid (β-cyfluthrin and λ-cyhalothrin) as the sole carbon source.

The bacterium responsible for the degradation of fluorinated pyrethroids was isolated using a 10-fold serial dilution method. The soil microbe consortium was diluted up to 10^−6^ times and then cultured on TSA plates. After overnight incubation, a total of eighteen colonies were observed. Each colony was inoculated into M9 minimal media supplemented with pyrethroid as the sole carbon source. Among the colonies, the MFK14 colony exhibited growth after overnight incubation, suggesting that this bacterium could utilise the fluorinated pyrethroids β-cyfluthrin and λ-cyhalothrin as carbon sources for growth, as indicated by the OD_600_ measurements shown in Figure 2.

**Figure 3:**
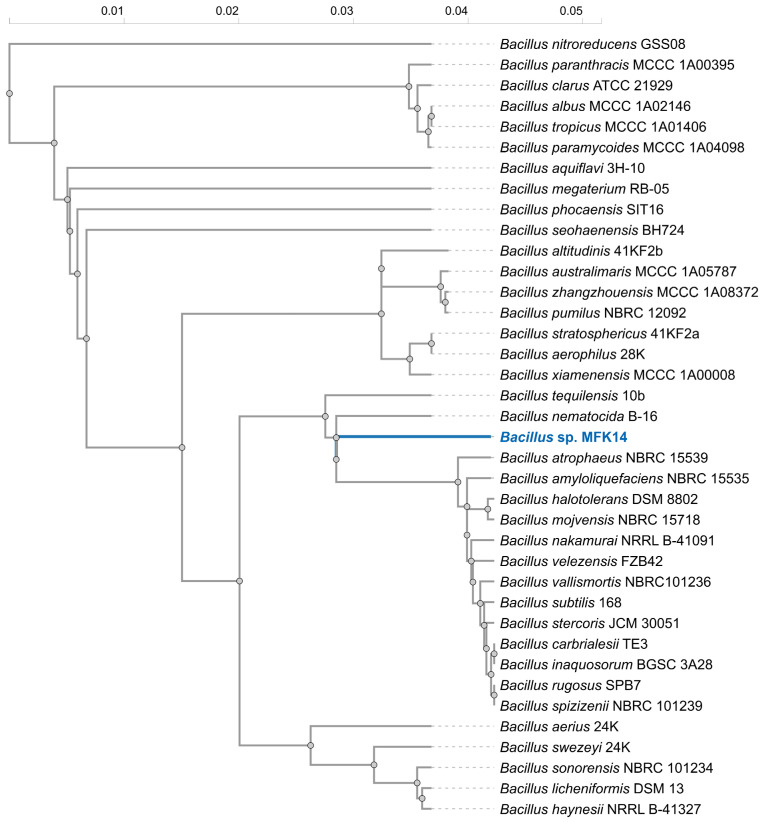
Phylogenetic analysis of isolated *Bacillus* sp. MFK14 using homologous 16S rRNA sequences from another *Bacillus* spp.

Whole genome sequencing confirmed that the isolated bacterium belongs to the genus *Bacillus* and was designated as *Bacillus* sp. MFK14. Phylogenetic analysis of 16S rRNA gene sequences revealed that *Bacillus* sp. MFK14 clusters closely with several species, including *B. nematocida*, *B. amyloliquefaciens*, *B. halotolerans*, and *B. subtilis*, suggesting a close evolutionary relationship within the genus (Figure 3). Notably, *B. nematocida* was identified as the closest phylogenetic neighbour, with a 16S rRNA sequence similarity of 97.2% to *Bacillus* sp. MFK14. Although this high similarity indicated a close genetic relationship, it falls within the range where a novel species could still be distinguished. Further taxonomic investigations, including phenotypic, chemotaxonomic, and genome-based analyses, are warranted to confirm its classification.

### 3.3. Metabolite Identification in Pyrethroid Degradation by Bacillus sp. MFK14

**Figure 4:**
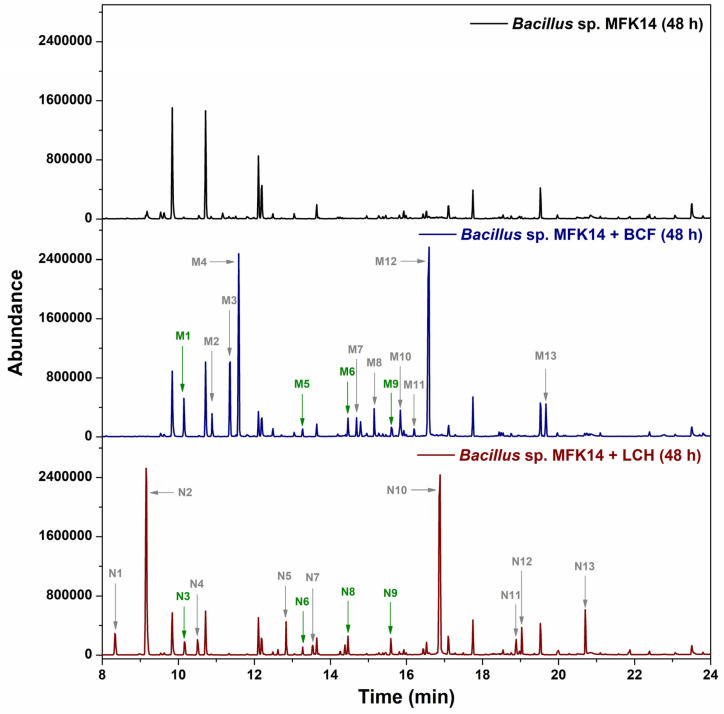
Total-ion chromatogram of *Bacillus* sp. MFK14 incubation with β-cyfluthrin (BCF) and λ-cyhalothrin (LCH) for 48 h. The control experiment was kept with bacteria without pyrethroids. The metabolites produced from β-cyfluthrin are numbered with “M”: M1–M13, and λ-cyhalothrin are numbered with “N”: N1–N13. Common metabolites are represented by green, whereas the specific metabolites of BCF and LCH are represented by grey.

To investigate the biodegradation of fluorinated pyrethroids, organic extracts from *Bacillus* sp. MFK14 cultures were analysed by GC–MS following MSTFA silylation. After 48 h of incubation with β-cyfluthrin and λ-cyhalothrin with *Bacillus* sp. MFK14, several degradation metabolites were identified which were absent in control samples (without any pyrethroids). Thirteen metabolites from β-cyfluthrin were labelled M1–M13 based on their retention times in the gas chromatogram, while thirteen metabolites from λ-cyhalothrin were labelled N1–N13 (Figure 4). The complete disappearance of the substrate peaks for β-cyfluthrin and λ-cyhalothrin after 48 h of incubation with *Bacillus* sp. MFK14 indicates that both compounds were fully degraded within this timeframe. This was confirmed by comparing the retention times of the authentic standards, with β-cyfluthrin observed at 23.45 min and λ-cyhalothrin at 22.54 min (Figure S1).

Metabolite identification was confirmed either through comparison with authentic standards or by matching the mass spectra with reference data from the NIST library. The mass spectra corresponding to each metabolite are provided in Supplementary Figures S2–S23. Among the identified metabolites, four were common to both pyrethroids: M1/N3 (hydroquinone) at 10.16 min, M5/N6 (hydroxyquinol) at 13.27 min, M6/N8 (4-hydroxy-6-oxohexa-2,4-dienoic acid) at 14.46 min, and M9/N9 (2-formyl-3-(hydroxymethyl)-2-methylcyclopropane-1-carboxylic acid) at 15.59 min. Both β-cyfluthrin and λ-cyhalothrin were extensively degraded by *Bacillus* sp. MFK14 over the incubation period, with distinct metabolic profiles for each compound (M-series and N-series metabolites). The chromatograms confirmed the bacterium’s capacity to metabolise fluorinated pyrethroids effectively, with no interference observed in the control sample.

**Figure 5:**
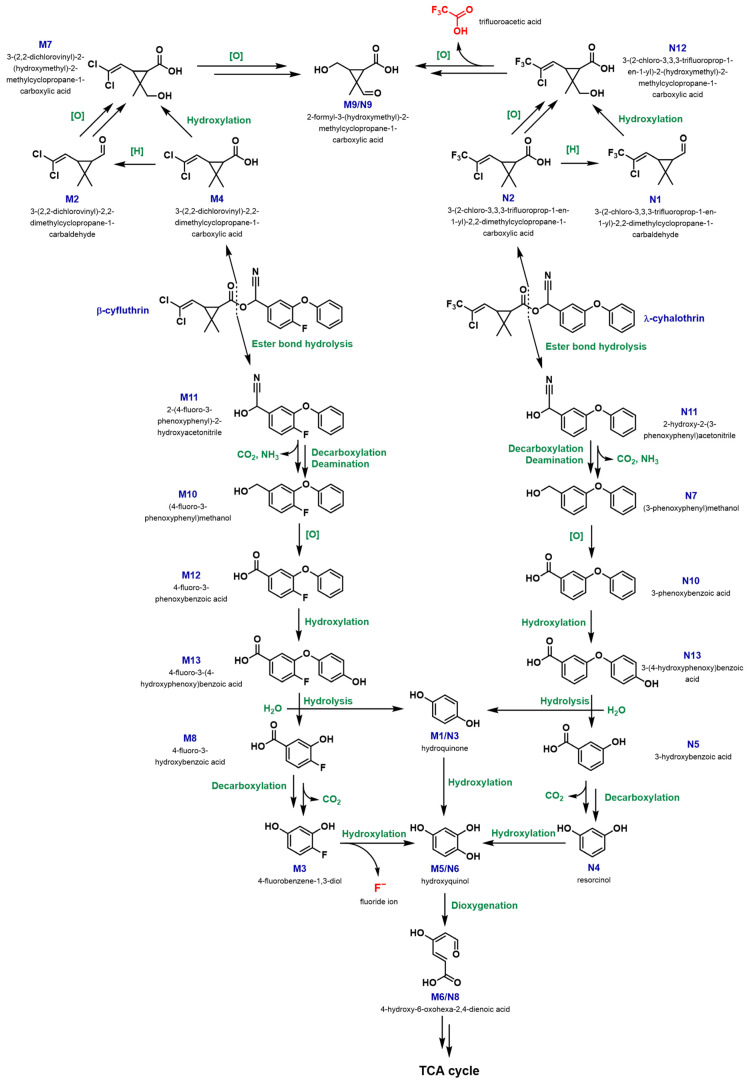
A common proposed pathway for biodegradation of fluorinated pyrethroid β-cyfluthrin and λ-cyhalothrin. The metabolites produced from β-cyfluthrin are numbered with “M”: M1–M13, and λ-cyhalothrin are numbered with “N”: N1–N13.

Figure 5 illustrates the proposed common pathway for the biodegradation of β-cyfluthrin and λ-cyhalothrin by *Bacillus* sp. MFK14. This figure summarises the complexity of the study, detailing metabolite structures, labels, names, and fluorinated end products, along with the types of reactions such as hydroxylation, oxidation, reduction, or decarboxylation.

#### 3.3.1. Biodegradation Pathway for β-cyfluthrin

In the biodegradation of β-cyfluthrin, key metabolites identified included M4 (3-(2,2-dichlorovinyl)-2,2-dimethylcyclopropane-1-carboxylic acid) at 11.59 min and M12 (4-fluoro-3-phenoxybenzoic acid) at 16.59 min, likely formed by cleavage of the central ester bond followed by oxidoreductive removal of the cyano (-CN) group. M4 was then converted into M7 (3-(2,2-dichlorovinyl)-2-(hydroxymethyl)-2-methylcyclopropane-1-carboxylic acid) via M2 (3-(2,2-dichlorovinyl)-2,2-dimethylcyclopropane-1-carbaldehyde) through reduction of the carboxyl group to an aldehyde, followed by oxidation and hydroxylation steps, or directly into M7 by a hydroxylation step. One of the end products, M9 (2-formyl-3-(hydroxymethyl)-2-methylcyclopropane-1-carboxylic acid), was formed from M7 through multiple steps of oxidative dechlorination. On the other hand, M12 was transformed into M13 (4-fluoro-3-(4-hydroxyphenoxy)benzoic acid) by hydroxylation, likely at the aromatic ring other than the fluorinated one. This was followed by hydrolysis of the diphenyl ether produced M1 (hydroquinone) and M8 (4-fluoro-3-hydroxybenzoic acid). M8 was subsequently converted to M5 (hydroxyquinol) via M3 (4-fluorobenzene-1,3-diol) through oxidative decarboxylation and defluorination, while M1 was directly transformed into M5 by hydroxylation to form hydroxyquinol. M5 underwent ring cleavage by a dioxygenation step to form M6 (4-hydroxy-6-oxohexa-2,4-dienoic acid), which was eventually catabolised through the TCA cycle.

#### 3.3.2. Biodegradation Pathway for λ-cyhalothrin

In the degradation of λ-cyhalothrin by *Bacillus* sp. MFK14, the major metabolites N2 (3-(2-chloro-3,3,3-trifluoroprop-1-en-1-yl)-2,2-dimethylcyclopropane-1-carboxylic acid) at 9.15 min and N10 (3-phenoxybenzoic acid) at 16.88 min were produced through a similar cleavage of the central ester bond and subsequent oxidoreductive removal of the -CN group. N2 was then converted into N12 (3-(2-chloro-3,3,3-trifluoroprop-1-en-1-yl)-2-(hydroxymethyl)-2-methylcyclopropane-1-carboxylic acid) via N1 (3-(2-chloro-3,3,3-trifluoroprop-1-en-1-yl)-2,2-dimethylcyclopropane-1-carbaldehyde) by reduction of the carboxyl group to an aldehyde, followed by oxidation and hydroxylation steps, or directly to N12 through hydroxylation. One of the end products, N9 (2-formyl-3-(hydroxymethyl)-2-methylcyclopropane-1-carboxylic acid), was formed from N12 through multiple steps of oxidative dechlorination. On the other hand, N10 was transformed into N13 (3-(4-hydroxyphenoxy)benzoic acid) by hydroxylation at one of the aromatic rings. Subsequent hydrolysis of the diphenyl ether produced N3 (hydroquinone) and N5 (3-hydroxybenzoic acid). N5 was then converted to N6 (hydroxyquinol) via N4 (resorcinol) through oxidative decarboxylation and hydroxylation, while N3 was directly transformed into N6 by hydroxylation to form hydroxyquinol. N6 underwent ring cleavage by a dioxygenation step to form N8 (4-hydroxy-6-oxohexa-2,4-dienoic acid), which was ultimately catabolised through the TCA cycle, like the pathway for β-cyfluthrin.

Furthermore, the phenolic metabolites M1/N3 (hydroquinone) at 10.16 min, N4 (resorcinol) at 10.50 min, and M5/N6 (hydroxyquinol) at 13.27 min were confirmed by comparing their retention times and mass spectra with those of authentic standards (Figure S24). These findings indicated the presence of key metabolites in the culture extracts, suggesting that *Bacillus* sp. MFK14 had the potential to biodegrade the fluorinated pyrethroids β-cyfluthrin and λ-cyhalothrin. The observed pathways appeared to involve the formation and subsequent transformation of phenolic compounds.

Water-soluble metabolites were identified by freeze-drying the culture supernatants of *Bacillus* sp. MFK14 was incubated with β-cyfluthrin and λ-cyhalothrin for 48 h. The dried residues were redissolved in D_2_O for ^19^F NMR analysis (Figure S25). Notably, fluoride ions (resonance at δ = −122.4 ppm) were detected in the β-cyfluthrin biodegradation sample, likely formed during the transformation of metabolite M3 to M5 through oxidative defluorination. However, trifluoroacetic acid (resonance at δ = −75.8 ppm) was detected in the λ-cyhalothrin biodegradation sample, possibly formed during the conversion of metabolite N12 to N9 via multiple steps of oxidative dechlorination and deacetylation. Both aqueous metabolites were confirmed against authentic standards, with NaF (resonance at δ = −122.4 ppm) and TFA (resonance at δ = −75.8 ppm) serving as references in the ^19^F NMR analysis. The detection of fluoride ions in the β-cyfluthrin sample indicated its mineralisation, while the presence of trifluoroacetic acid, although resistant to microbial degradation, suggested that λ-cyhalothrin has been effectively catabolised into a smaller fluorinated end-product. The comprehensive degradation pathway indicates the potential of *Bacillus* sp. MFK14 in breaking down persistent fluorinated pesticides, indicating its relevance for environmental bioremediation efforts.

### 3.4. CYP Involvement in the Biodegradation of β-cyfluthrin and λ-cyhalothrin

The role of cytochrome P450 enzymes (CYPs) in the biodegradation of β-cyfluthrin and λ-cyhalothrin by *Bacillus* sp. MFK14 was evaluated using the CYP inhibitor 1-aminobenzotriazole (1-ABT). To assess this, CYPs were inhibited by pre-incubating *Bacillus* sp. MFK14 with 1-ABT for one hour before adding the pesticides. These cultures were then used for biodegradation experiments.

**Figure 6:**
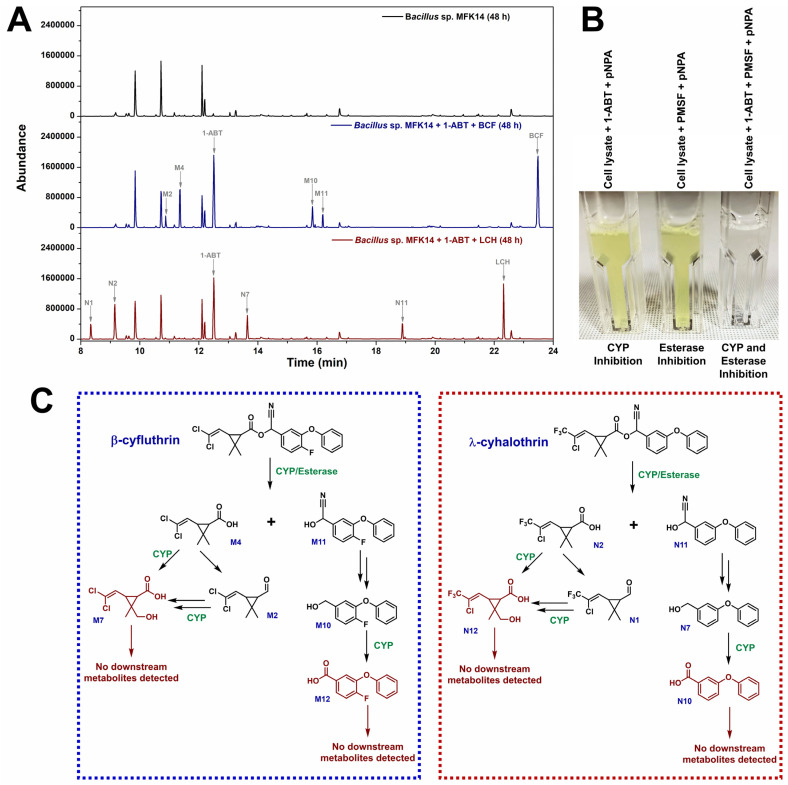
(**A**) Total ion chromatogram illustrating the effect of CYP inhibition (using 1 mM 1-ABT) on the biodegradation of β-cyfluthrin (BCF) and λ-cyhalothrin (LCH) by *Bacillus* sp. MFK14 after 48 h of incubation. (**B**) Enzyme inhibition assay using 1 mM 1-ABT (CYP inhibitor) and 1 mM PMSF (esterase inhibitor) to demonstrate ester bond hydrolysis in the cell lysate of *Bacillus* sp. MFK14, with p-NPA as the substrate. This experiment demonstrates that both CYP and esterase independently exhibit ester bond hydrolysis. (**C**) Proposed role of CYPs in various steps of the biodegradation pathways of β-cyfluthrin (in the blue box) and λ-cyhalothrin (in the red box).

GC–MS analysis revealed that most metabolites were undetected following CYP inhibition, except for early-stage degradation products. In the β-cyfluthrin biodegradation pathway, metabolites such as M2 (3-(2,2-dichlorovinyl)-2,2-dimethylcyclopropane-1-carbaldehyde), M4 (3-(2,2-dichlorovinyl)-2,2-dimethylcyclopropane-1-carboxylic acid), M10 (4-fluoro-3-phenoxyphenyl)methanol), and M11 (2-(4-fluoro-3-phenoxyphenyl)-2-hydroxyacetonitrile) were still detected (Figure 6A). The presence of M4 and M11, formed immediately after ester bond hydrolysis, indicated that esterases facilitated this step, even in the presence of the CYP inhibitor. However, the substantial peak of the parent compound β-cyfluthrin (at 23.45 min) after 48 h, compared to normal biodegradation without 1-ABT (Figure 6), suggested that CYP inhibition slowed the rate of ester bond hydrolysis. Further transformations of M4 to M2 and M11 to M10, which do not require CYPs, were observed. However, the degradation of M2 and M10—steps solely dependent on CYPs—was inhibited, preventing the formation of downstream metabolites such as M1, M3, M5, M6, M7, M8, M9, M12, and M13.

Similarly, in the biodegradation of λ-cyhalothrin, early-stage metabolites such as N1 (3-(2-chloro-3,3,3-trifluoroprop-1-en-1-yl)-2,2-dimethylcyclopropane-1-carbaldehyde), N2 (3-(2-chloro-3,3,3-trifluoroprop-1-en-1-yl)-2,2-dimethylcyclopropane-1-carboxylic acid), N7 ((3-phenoxyphenyl)methanol), and N11 (2-hydroxy-2-(3-phenoxyphenyl)acetonitrile) were detected (Figure 6A). This suggested that esterases were responsible for the initial ester bond hydrolysis, even when CYPs were inhibited. However, the significant persistence of λ-cyhalothrin (at 22.54 min) after 48 h, along with the absence of further degradation of N1 and N7, indicated that CYP inhibition slowed the biodegradation process, preventing the formation of downstream metabolites such as N3, N4, N5, N6, N8, N9, N10, N12, and N13.

To further confirm the involvement of cytochrome P450 enzymes (CYPs) and esterases in ester bond hydrolysis, a separate enzyme inhibition assay was performed using *Bacillus* sp. MFK14 cell-free lysate. Three reactions were set up. In the first reaction, the lysate was incubated with 1-ABT to inhibit CYPs; in the second reaction, the lysate was treated with PMSF to inhibit esterases; and in the third reaction, the lysate was treated with both 1-ABT and PMSF to achieve dual inhibition (Figure 6B). After 10 min of incubation, ester bond hydrolysis was assessed using p-NPA as a substrate, which releases p-nitrophenol (p-NP) upon ester bond hydrolysis, producing a yellow colour. The reaction with 1-ABT and p-NPA developed a yellow colour, indicating that esterases were active in cleaving the ester bond. Similarly, the reaction with PMSF and p-NPA also produced a yellow colour, suggesting that CYPs were involved in ester bond hydrolysis. However, the reaction with both 1-ABT and PMSF did not produce any yellow colour, confirming that both CYPs and esterases were required for ester bond hydrolysis. These results confirm that both CYPs and esterases are essential for ester bond hydrolysis of p-NPA. Similarly, in the biodegradation of β-cyfluthrin and λ-cyhalothrin, both enzyme systems (CYPs and esterases) were involved in the initial cleavage of ester bonds, with CYPs playing a key role in the subsequent degradation steps.

Figure 6C illustrates the proposed role of *Bacillus* sp. MFK14 CYPs in various steps of the biodegradation pathways of β-cyfluthrin and λ-cyhalothrin. For β-cyfluthrin, CYPs facilitated transformations such as β-cyfluthrin to M4 and M11, M4 to M7, M2 to M7, and M10 to M12. In λ-cyhalothrin biodegradation, CYPs mediated the conversion of λ-cyhalothrin to N11 and N2, N2 to N12, N1 to N12, and N7 to N10. Fluorinated metabolites resulting from the biodegradation of β-cyfluthrin included fluoride ions, while trifluoroacetic acid was produced from λ-cyhalothrin. This production was likely mediated by multiple enzymes, with CYPs being a potential contributor to the process. Esterases play a crucial role in the initial breakdown of β-cyfluthrin into M4 and M11 and λ-cyhalothrin into N11 and N2 by cleaving the ester bonds. While esterase activity is independent, it worked in conjunction with CYPs, which also contributed to ester bond hydrolysis, complementing the overall biodegradation process.

### 3.5. Predicted CYPome of Bacillus sp. MFK14

To explore the uncharacterised genome of *Bacillus* sp. MFK14, a search for CYP gene sequences was conducted to support the inhibition experiments. Using tBLASTn, CYP orthologs were identified to predict the complete CYP enzyme set, or “CYPome,” as listed in Table 1. In silico analysis revealed a high degree of conservation among CYP genes in closely related species, including *B. subtilis*, *B. cereus*, *B. anthracis*, *B. weihenstephanensis*, *B. megaterium*, and *B. licheniformis*. Notably, the identified CYPs in *Bacillus* sp. MFK14 included variants of CYP102, CYP106, CYP107, CYP109, CYP134, CYP152, and CYP197. The putative gene sequences for these CYPs, extracted from the newly sequenced genome of *Bacillus* sp. MFK14 are provided in Supplementary Table S1. Among these, the CYPs of *B. subtilis* exhibited high sequence identity (above 90%) with the identified CYPs in *Bacillus* sp. MFK14, except for CYP109A1 (only 42.1% identity). The genomic evidence confirmed the presence of CYP genes in *Bacillus* sp. MFK14, indicating their crucial role in the biodegradation of β-cyfluthrin and λ-cyhalothrin. These findings align with the inhibition experiments, which showed CYPs are essential for degrading these pesticides, highlighting their importance in environmental cleanup.

## 4. Discussion

The use of pyrethroids in agriculture practices for pest control leads to environmental pollution, negatively impacting human health and reducing soil microbial populations, which diminishes soil fertility [17]. Some soil bacteria can tolerate these harmful metabolites, evolving mechanisms to degrade pyrethroids [18]. In this study, we utilised a bacterial consortium isolated from garden soil to degrade the fluorinated pyrethroids β-cyfluthrin and λ-cyhalothrin. Similar studies, such as Birolli et al., Liu et al., and Zhang et al., have demonstrated the degradation of various pyrethroids using bacterial consortia from different environments [19,20,21]. Our study found that newly isolated *Bacillus* sp. MFK14 from garden soil completely degraded the β-cyfluthrin and λ-cyhalothrin within 48 h, producing 13 metabolites in each pesticide. In comparison, previous research reported that *Bacillus subtilis* BSF01 degraded 89.4% of 50 mg/L β-cypermethrin in 7 days [9], *Brevibacterium aureum* DG-12 achieved 88.6% degradation of 50 mg/L β-cyfluthrin within 5 days [22], and *Bacillus thuringiensis* ZS-19 completely degraded 100 mg/L of λ-cyhalothrin in 72 h [23]. The degradation efficiency of λ-cyhalothrin and β-cyfluthrin in bacteria and fungi is summarised in Table 2, comparing the findings of this study with previously reported microbial strains.

Esterases play a vital role in pyrethroid degradation by cleaving the primary ester bonds in these compounds [29]. A carboxylesterase from *Bacillus cereus* BCC01, which functioned at pH 8 and 30 °C, was characterised for its pyrethroid-degrading activity [30]. Similarly, a cypermethrin-degrading esterase from *Bacillus subtilis* was most effective at pH 7.0 and 32 °C [31]. However, beyond esterase activity, our study also demonstrated that the degradation of β-cyfluthrin and λ-cyhalothrin involved cytochrome P450 (CYP)-mediated cleavage of ester bonds in *Bacillus* sp. MFK14. This was confirmed using 1-ABT, a CYP inhibitor, which showed that β-cyfluthrin was transformed into M4 and M11, while λ-cyhalothrin was converted to N11 and N2 through CYP-mediated biotransformation. Khan and Murphy previously identified similar ester bond hydrolysis in transfluthrin and β-cyfluthrin by *Cunninghamella* spp. (*C. elegans*, *C. blakesleeana*, and *C. echinulata*), attributing this activity to cytochrome P450s (CYPs) [10]. Further research revealed that CYP5208A3 and CYP5313D1, along with their redox partner CYP reductase (CPR_C) from *C. elegans*, were involved in the oxidative ester cleavage of the transfluthrin pyrethroid [10]. Additionally, Hedges et al. demonstrated significant variability in the activity of recombinant human CYPs across different pyrethroids (including λ-cyhalothrin, bifenthrin, and β-cyfluthrin), with CYP2C19 showing the highest effectiveness [32]. Our study further revealed that CYPs in *Bacillus* sp. MFK14 not only cleaved ester bonds but also participated in the transformation of various metabolites in β-cyfluthrin and λ-cyhalothrin biodegradation. Specifically, β-cyfluthrin was transformed through M4 to M7, M2 to M7, and M10 to M12, while λ-cyhalothrin was converted from N2 to N12, N1 to N12, and N7 to N10. These conversions were supported by CYP inhibition experiments (Figure 6C). In silico analysis of the CYPome in *Bacillus* sp. MFK14 genome revealed a significant number of CYP genes (23 variants), indicating a high potential for CYPs to participate in ester hydrolysis, as well as monooxygenation, defluorination, and other reactions during pyrethroid biodegradation.

The presence of fluoride ions in β-cyfluthrin and trifluoroacetic acid in λ-cyhalothrin following 48 h of incubation with *Bacillus* sp. MFK14 was confirmed by ^19^F-NMR analysis of the aqueous fraction. The release of fluoride ions likely occurred during the conversion of 4-fluorobenzene-1,3-diol (M3) to hydroxyquinol (M5) in β-cyfluthrin biodegradation, potentially catalysed by cytochrome P450s (CYPs), as previously described by Harkey et al. in the CYP-mediated oxidative defluorination of 4-fluorophenol to hydroquinone [33]. Similarly, Li et al. observed defluorinated metabolites in the bacterial degradation of β-cyfluthrin [7].

Dechlorination of the 2,2-dichlorovinyl group in β-cyfluthrin (conversion from M7 to M9) and the 2-chloro-3,3,3-trifluoroprop-1-en-1-yl group in λ-cyhalothrin (conversion from N12 to N9) was rare, with limited reports documenting these dechlorinated metabolites. For example, Birolli et al. demonstrated that a *Bacillus* sp. CSA-1 consortium of 10 bacterial strains achieved rapid CYP-mediated biodegradation of cypermethrin, identifying dechlorinated metabolites via LC-MS/MS analysis [19]. Additionally, Li et al. identified a dechlorinated metabolite, (2,2,3,3-tetramethyl-cyclopropyl)-methanol, resulting from the removal of terminal chlorines during CYP-mediated β-cyfluthrin degradation by a bacterial consortium [7]. Zhang et al. reviewed the dechlorination of 2,2-dichlorovinyl groups in dichlorvos by various microorganisms, including *Pseudomonas*, *Bacillus*, *Fusarium*, *Aspergillus*, and *Trichoderma*. Consequently, the dechlorination of the 2-chloro-3,3,3-trifluoroprop-1-en-1-yl group in λ-cyhalothrin may have resulted in the release of trifluoroacetic acid, as identified in our ^19^F-NMR analysis [34].

In addition to CYPs, the degradation processes for both pyrethroids involved a possible range of enzymes, including hydrolases, dioxygenases, etc. Earlier work by Schmidt et al. described the nonspecific attack of dioxygenase on halodiphenyl ethers, leading to the formation of halophenols during biodegradation by *Sphingomonas* sp. strain SS3 [35]. This mechanism is commonly observed in both bacterial and fungal degradation [36,37]. In our study, *Bacillus* sp. MFK14 exhibited a similar diphenyl ether moiety degradation pattern in both β-cyfluthrin and λ-cyhalothrin. Following diphenyl ether cleavage, the phenolic metabolites hydroquinone (M1/N3) at 10.16 min, resorcinol (N4) at 10.50 min, and hydroxyquinol (M5/N6) at 13.27 min were identified by comparing their retention times and mass spectra with those of authentic standards. These findings provide strong evidence for the production of these phenolic metabolites during the biodegradation of β-cyfluthrin and λ-cyhalothrin by *Bacillus* sp. MFK14.

The genome analysis of *Bacillus* sp. MFK14 also revealed the presence of catechol-2,3-dioxygenase with high sequence identity (99%) to *Bacillus subtilis* strain 168 catechol-2,3-dioxygenase (Protein ID: P54721). This supported the observed ring cleavage from hydroxyquinol (M5/N6) to 4-hydroxy-6-oxohexa-2,4-dienoic acid (M6/N8) in both pesticides, which was subsequently catabolised through the TCA cycle. In summary, the proposed biodegradation pathways for β-cyfluthrin and λ-cyhalothrin by *Bacillus* sp. MFK14 illustrated the complex interplay of various enzymes in breaking down these fluorinated pyrethroids. Understanding these pathways enhanced our knowledge of microbial degradation mechanisms and could have informed strategies for environmental remediation.

## 5. Conclusions

This study highlighted the crucial role of cytochrome P450 enzymes in the biodegradation of fluorinated pyrethroids by *Bacillus* sp. MFK14, a newly isolated strain from garden soil. The strain effectively degraded β-cyfluthrin and λ-cyhalothrin, releasing fluoride ions and trifluoroacetic acid (TFA), demonstrating the potential of microbial bioremediation to mitigate the environmental impact of these persistent insecticides. Notably, *Bacillus* sp. MFK14 completely degraded both β-cyfluthrin and λ-cyhalothrin within 48 h at 30 °C, showcasing its efficiency in degrading these hazardous compounds. The findings also revealed that, despite differing fluorinations, β-cyfluthrin and λ-cyhalothrin shared degradation pathways, showcasing the adaptability and specificity of bacterial CYP enzymes in breaking down complex compounds. Inhibition studies with 1-ABT further confirm the involvement of CYP enzymes in this process, while genomic analysis of *Bacillus* sp. MFK14′s CYPome showed significant sequence similarity with known bacterial CYPs, highlighting the evolutionary conservation and functional importance of these enzymes.

## 6. Future Perspectives

Microbial degradation offers an eco-friendly, cost-effective solution for pesticide, herbicide, and insecticide remediation, with advantages over traditional methods by breaking down pollutants into less toxic forms. It can be applied in situ, reducing environmental persistence and preserving soil and water quality. However, its efficiency can vary with environmental conditions, and challenges such as toxic intermediates and slow microbial adaptation to synthetic pollutants exist. Genetic engineering and optimising microbial consortia could improve the process and enhance its sustainability. In line with these considerations, the environmental challenges posed by fluorinated pyrethroids make this research a promising foundation for developing biotechnological applications aimed at reducing their persistence and toxicity. Future studies should focus on the genetic engineering of *Bacillus* sp. MFK14 and heterologous expression of predicted CYPs to enhance degradation efficiency. Moreover, investigating the interaction of these bacterial CYPs with other classes of persistent organic pollutants could broaden the scope of bioremediation strategies, ultimately contributing to more sustainable agricultural practices.

## Figures and Tables

**Table 1:** CYPs in *Bacillus* sp. MFK14 and their sequence similarity to orthologs in other *Bacillus* spp.

CYP Name	Protein ID *	Source Organism	Protein Identity of CYP Orthologs (%) in *Bacillus* sp. MFK14
CYP102A1	P14779	*Bacillus megaterium*	65.0
CYP102A2	O08394	*Bacillus subtilis*	98.5
CYP102A3	O08336	*Bacillus subtilis*	99.3
CYP102A4	A0A6L7HJT4	*Bacillus anthracis*	75.2
CYP102A5	Q81BF4	*Bacillus cereus*	75.5
CYP102A7	NA	*Bacillus licheniformis*	61.8
CYP102A8	NA	*Bacillus thuringiensis*	75.8
CYP102A9	NA	*Bacillus weihenstephanensis*	75.0
CYP102A15	NA	*Bacillus pumilus*	60.7
CYP106A1	P14762	*Bacillus megaterium*	40.8
CYP106A2	Q06069	*Bacillus megaterium*	42.0
CYP106B1	A0A6L7HJD6	*Bacillus anthracis*	47.1
CYP107DY1	D5E3H2	*Bacillus subtilis*	42.8
CYP107H1	P53554	*Bacillus subtilis*	98.9
CYP107J1	O08469	*Bacillus subtilis*	94.6
CYP107J2	A0A6L7H515	*Bacillus anthracis*	57.6
CYP107J3	Q81CY0	*Bacillus cereus*	57.6
CYP107K1	O31785	*Bacillus subtilis*	98.9
CYP109A1	P27632	*Bacillus subtilis*	42.1
CYP109B1	O34374	*Bacillus subtilis*	93.0
CYP134A1	O34926	*Bacillus subtilis*	96.8
CYP152A1	O31440	*Bacillus subtilis*	95.2
CYP197A1	Q9KFA6	*Bacillus halodurans*	27.7

* NA—protein ID is not available in the UniProt, but its sequence is available in the Cytochrome P450 database (https://drnelson.uthsc.edu/bacteria/; accessed on 10 January 2025).

**Table 2:** Comparison of β-cyfluthrin and λ-cyhalothrin degradation by different microorganisms.

Microorganism	Type	Concentration (mg/L)	Degradation (%)	Time (days)	Temperature (°C)	Reference
**β-Cyfluthrin**						
***Bacillus* sp. MFK14**	**Bacteria**	**100**	**100**	**2**	**30**	**Current study**
*Photobacterium ganghwense* 6046	Bacteria	100	82.8	3	30	[24]
*Brevibacterium aureum* DG-12	Bacteria	50	88.6	5	27	[22]
*Lysinibacillus sphaericus* FLQ-11-1	Bacteria	50	80.4	5	35	[25]
*Cunninghamella* spp.	Fungus	100	NA ^1^	5	28	[10]
**λ-cyhalothrin**						
***Bacillus* sp. MFK14**	**Bacteria**	**100**	**100**	**2**	**30**	**Current study**
*Bacillus thuringiensis* ZS-19	Bacteria	100	100	3	30	[23]
*Paracoccus acridae* SCU-M53	Bacteria	75	79.84	2	28	[26]
*Cunninghamella elegans* DSM1908	Fungus	100	NA ^1^	5	28	[27]
*Aspergillus* sp. CBMAI 1829	Fungus	100	44.8	14	32	[28]

^1^ NA = not available.

## Data Availability

Data will be made available on request.

## Supplementary Materials

The following supporting information can be downloaded at https://www.mdpi.com/article/10.3390/jox15020058/s1: Figure S1: GC-MS analysis of standards: (A) β-cyfluthrin, and (B) λ-cyhalothrin; Figure S2: Mass spectrum of metabolite M1 or N3 (t_R_ = 10.16 min; M^+^ = 254); Figure S3: Mass spectrum of metabolite M2 (t_R_ = 10.91 min; M^+^ = 192); Figure S4: Mass spectrum of metabolite M3 (t_R_ = 11.36 min; M^+^ = 272 (257+15)); Figure S5: Mass spectrum of metabolite M4 (t_R_ = 11.59 min; M^+^ = 280 (265+15)); Figure S6: Mass spectrum of metabolite M5 or N6 (t_R_ = 13.27 min; M^+^ = 342); Figure S7: Mass spectrum of metabolite M6 or N8 (t_R_ = 14.46 min; M^+^ = 286); Figure S8: Mass spectrum of metabolite M7 (t_R_ = 14.69 min; M^+^ = 368 (353+15)); Figure S9: Mass spectrum of metabolite M8 (t_R_ = 15.14 min; M^+^ = 300); Figure S10: Mass spectrum of metabolite M9 or N9 (t_R_ = 15.59 min; M^+^ = 302); Figure S11: Mass spectrum of metabolite M10 (t_R_ = 15.85 min; M^+^ = 290); Figure S12: Mass spectrum of metabolite M11 (t_R_ = 16.19 min; M^+^ = 315); Figure S13: Mass spectrum of metabolite M12 (t_R_ = 16.59 min; M^+^ = 304); Figure S14: Mass spectrum of metabolite M13 (t_R_ = 19.66 min; M^+^ = 392); Figure S15: Mass spectrum of metabolite N1 (t_R_ = 8.33 min; M^+^ = 226); Figure S16: Mass spectrum of metabolite N2 (t_R_ = 9.15 min; M^+^ = 314 (299+15)); Figure S17: Mass spectrum of metabolite N4 (t_R_ = 10.50 min; M^+^ = 254); Figure S18: Mass spectrum of metabolite N5 (t_R_ = 12.83 min; M^+^ = 282); Figure S19: Mass spectrum of metabolite N7 (t_R_ = 13.53 min; M^+^ = 272); Figure S20: Mass spectrum of metabolite N10 (t_R_ = 16.88 min; M^+^ = 286); Figure S21: Mass spectrum of metabolite N11 (t_R_ = 18.88 min; M^+^ = 297); Figure S22: Mass spectrum of metabolite N12 (t_R_ = 19.03 min; M^+^ = 402 (387+15)); Figure S23: Mass spectrum of metabolite N13 (t_R_ = 20.70 min; M^+^ = 374); Figure S24: GC-MS analysis of standards: (A) resorcinol, (B) hydroquinone, and (C) hydroxyquinol; Figure S25: ^19^F-NMR analysis confirmed the presence of (A) fluoride ion in β-cyfluthrin, and (B) trifluoroacetic acid in λ-cyhalothrin after 48 h incubation with *Bacillus* sp. MFK14; Table S1: Putative CYP sequences were extracted from the newly sequenced genome of *Bacillus* sp. MFK14.

## Abbreviations

The following abbreviations are used in this manuscript:

1-ABT	1-aminobenzotriazole
^19^F NMR	^19^F nuclear magnetic resonance spectroscopy
BCF	β-cyfluthrin
LCH	λ-cyhalothrin
CYP	Cytochrome P450
GC-MS	Gas chromatography-mass spectrometry
PBS	Phosphate-buffered saline
p-NPA	p-Nitrophenyl acetate
PMSF	Phenylmethylsulfonyl fluoride
TFA	Trifluoroacetic acid
TSB	Tryptone soya broth
TSA	Tryptone soya agar
