# Supplementary material for: Bacterial Cytochrome P450 Involvement in the Biodegradation of Fluorinated Pyrethroids
Source: J Xenobiot. 2025 Apr 18;15(2):58. doi: 10.3390/jox15020058 (PMC12028412; doi:10.3390/jox15020058)
Supplement: Supplementary file 1 [file jox-15-00058-s001.zip › jox-3526876-supplementary.pdf]

Supplementary Materials

Bacterial cytochrome P450 involvement in the biodegradation of fluorinated pyrethroids

Mohd Faheem Khan [1*], Jun Liao [1,2], Zhenyang Liu [1,2] and Gaurav Chugh [3]

[1]School of Agriculture and Food Science, University College Dublin, Belfield, Dublin 4, Ireland

[2] Guangzhou Dublin International College of Life Sciences & Technology, South China Agricultural University, Guangzhou, Guangdong, China

[3] School of Biomolecular and Biomedical Science, University College Dublin, Belfield, Dublin 4, Ireland

* Corresponding authors: mohd.khan@ucd.ie

Figure Legends

Figure S1: GC-MS analysis of standards: (A) β-cyfluthrin, and (B) λ-cyhalothrin.

Figure S2: Mass spectrum of metabolite M1 or N3 (t_R = 10.16 min; M^+ = 254).

Figure S3: Mass spectrum of metabolite M2 (t_R = 10.91 min; M^+ = 192).

Figure S4: Mass spectrum of metabolite M3 (t_R = 11.36 min; M^+ = 272 (257+15)).

Figure S5: Mass spectrum of metabolite M4 (t_R = 11.59 min; M^+ = 280 (265+15)).

Figure S6: Mass spectrum of metabolite M5 or N6 (t_R = 13.27 min; M^+ = 342).

Figure S7: Mass spectrum of metabolite M6 or N8 (t_R = 14.46 min; M^+ = 286).

Figure S8: Mass spectrum of metabolite M7 (t_R = 14.69 min; M^+ = 368 (353+15)).

Figure S9: Mass spectrum of metabolite M8 (t_R = 15.14 min; M^+ = 300).

Figure S10: Mass spectrum of metabolite M9 or N9 (t_R = 15.59 min; M^+ = 302).

Figure S11: Mass spectrum of metabolite M10 (t_R = 15.85 min; M^+ = 290).

Figure S12: Mass spectrum of metabolite M11 (t_R = 16.19 min; M^+ = 315).

Figure S13: Mass spectrum of metabolite M12 (t_R = 16.59 min; M^+ = 304).

Figure S14: Mass spectrum of metabolite M13 (t_R = 19.66 min; M^+ = 392).

Figure S15: Mass spectrum of metabolite N1 (t_R = 8.33 min; M^+ = 226).

Figure S16: Mass spectrum of metabolite N2 (t_R = 9.15 min; M^+ = 314 (299+15)).

Figure S17: Mass spectrum of metabolite N4 (t_R = 10.50 min; M^+ = 254).

Figure S18: Mass spectrum of metabolite N5 (t_R = 12.83 min; M^+ = 282).

Figure S19: Mass spectrum of metabolite N7 (t_R = 13.53 min; M^+ = 272).

Figure S20: Mass spectrum of metabolite N10 (t_R = 16.88 min; M^+ = 286).

Figure S21: Mass spectrum of metabolite N11 (t_R = 18.88 min; M^+ = 297).

Figure S22: Mass spectrum of metabolite N12 (t_R = 19.03 min; M^+ = 402 (387+15)).

Figure S23: Mass spectrum of metabolite N13 (t_R = 20.70 min; M^+ = 374).

Figure S24: GC-MS analysis of standards: (A) resorcinol, (B) hydroquinone, and (C) hydroxyquinol.

Figure S25: ^{19}F-NMR analysis confirmed the presence of (A) fluoride ion in β-cyfluthrin; and (B) trifluoroacetic acid in λ-cyhalothrin after 48 h incubation with *Bacillus* sp. MFK14.

Table Legends

Table S1: Putative CYP sequence from extracted from newly sequenced genome of *Bacillus* sp. MFK14

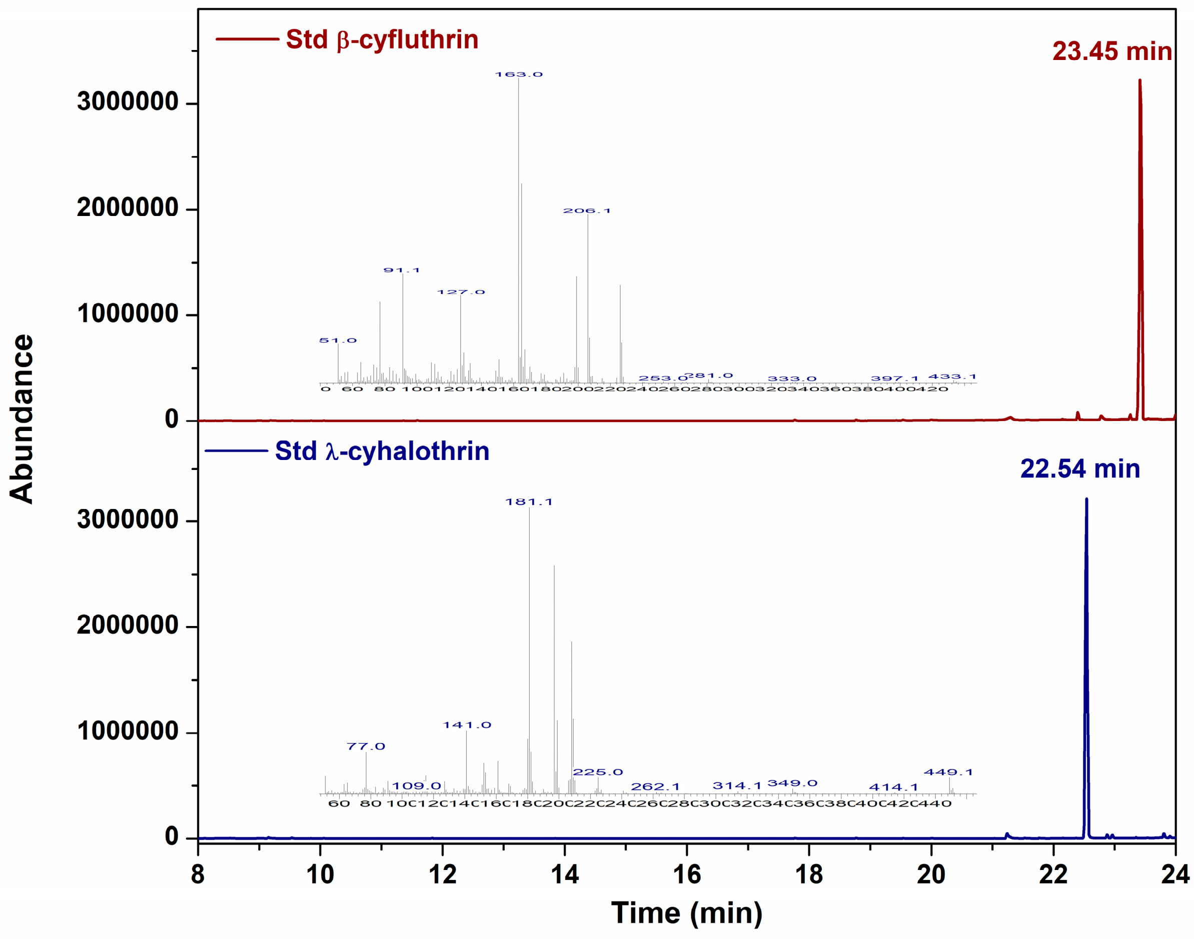

Figure S1: GC-MS analysis of standards: (A) β-cyfluthrin, and (B) λ-cyhalothrin.

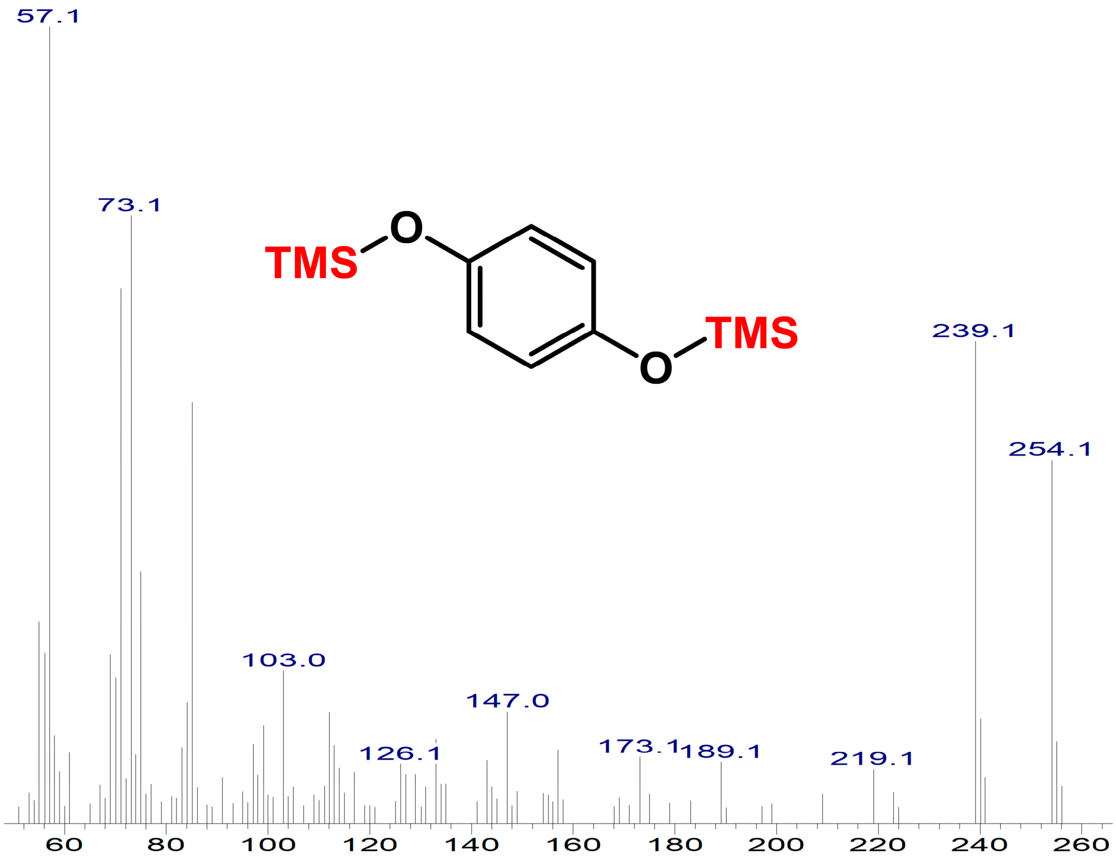

Figure S2: Mass spectrum of metabolite M1 or N3 (t_R = 10.16 min; M^+ = 254).

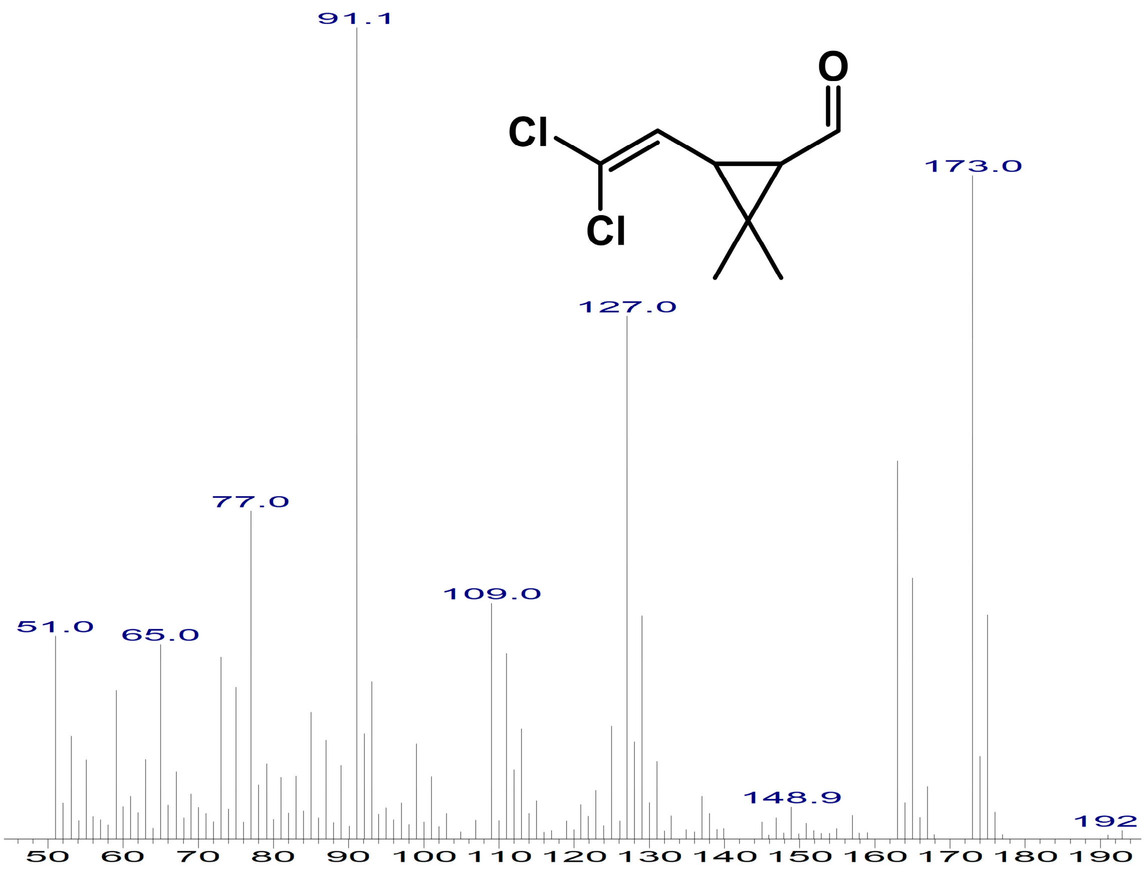

Figure S3: Mass spectrum of metabolite M2 (t_R = 10.91 min; M^+ = 192).

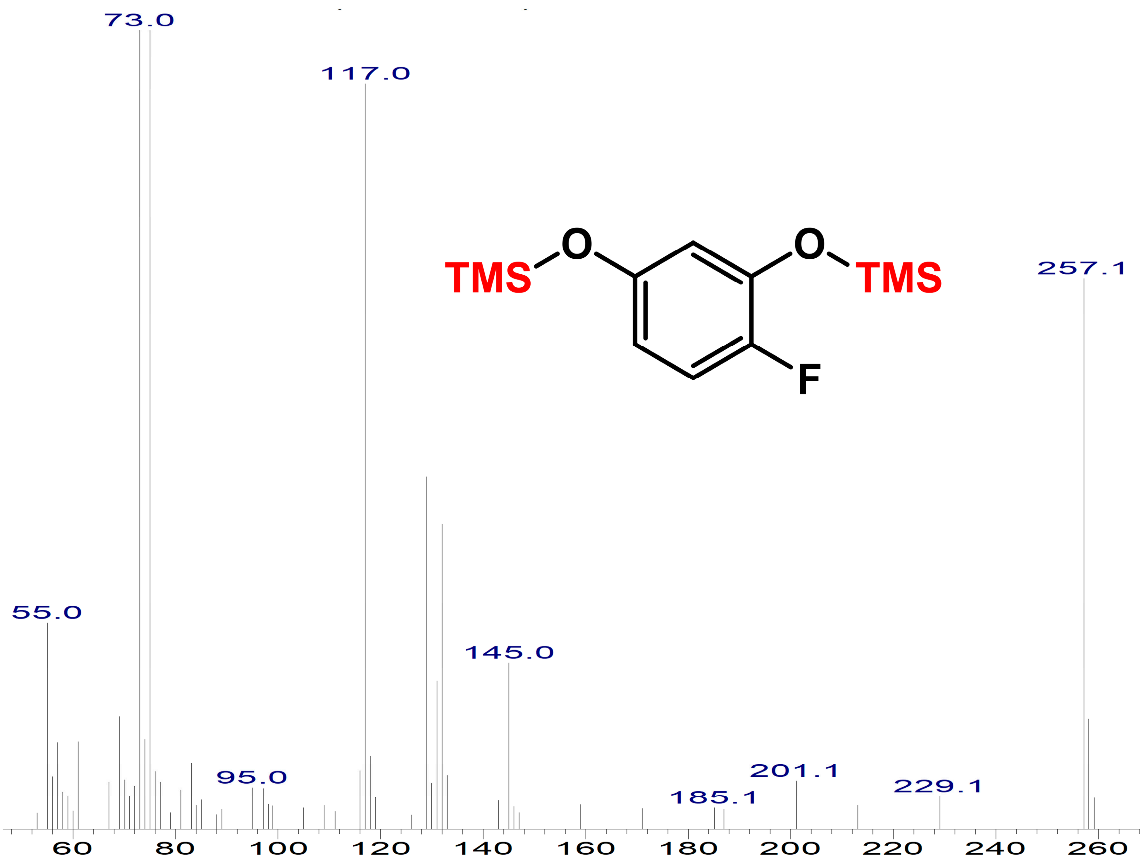

Figure S4: Mass spectrum of metabolite M3 (t_R = 11.36 min; M^+ = 272 (257+15)).

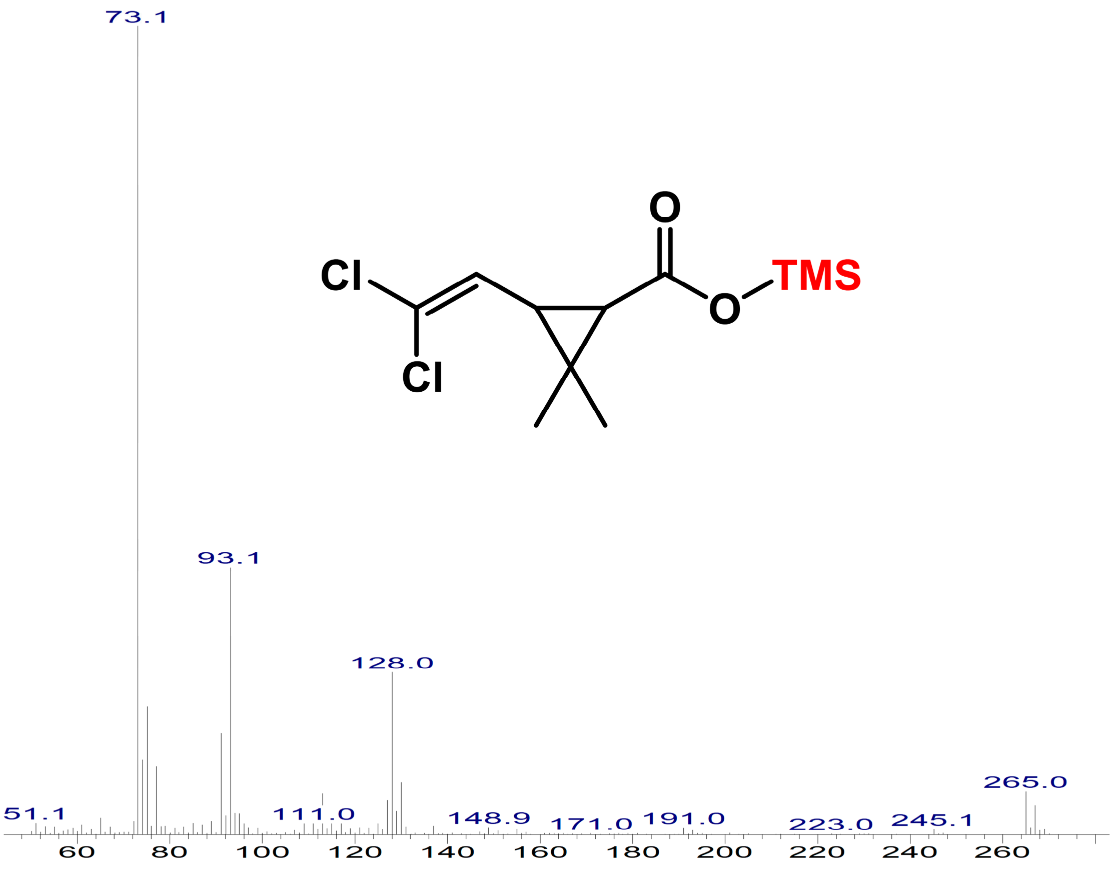

Figure S5: Mass spectrum of metabolite M4 (t_R = 11.59 min; M^+ = 280 (265+15)).

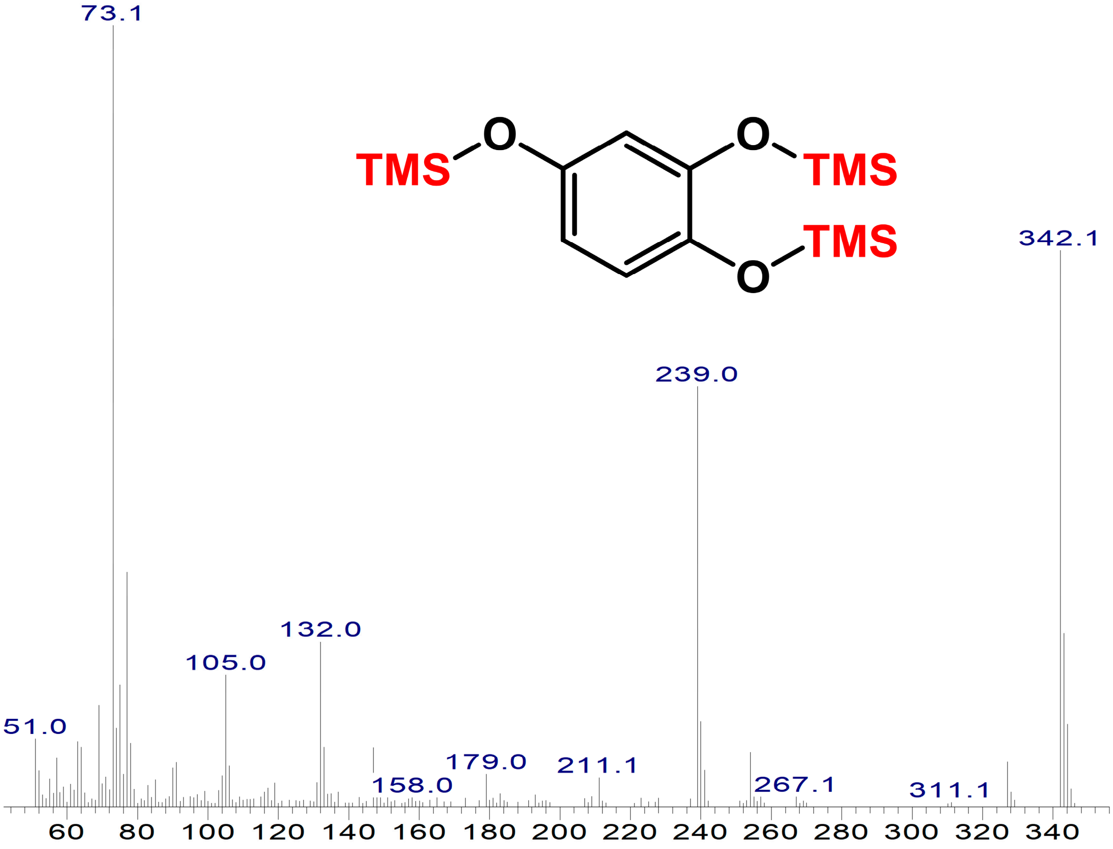

Figure S6: Mass spectrum of metabolite M5 or N6 (t_R = 13.27 min; M^+ = 342).

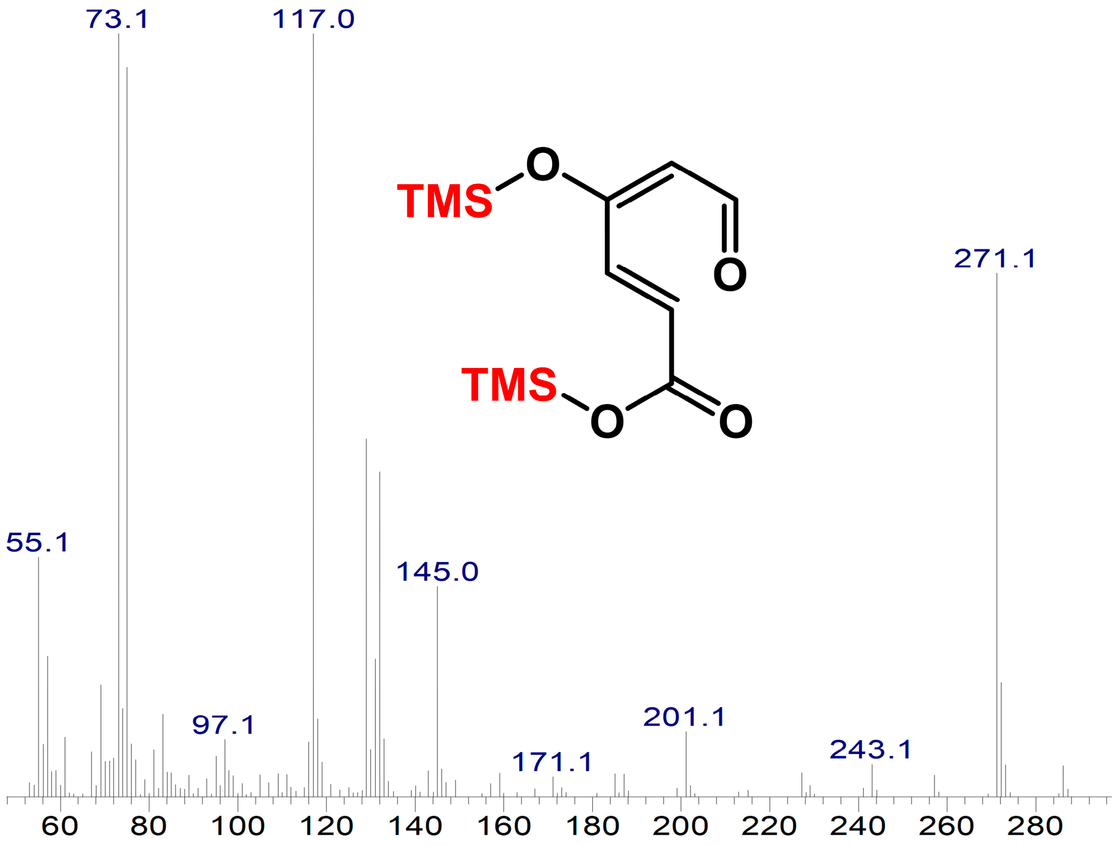

Figure S7: Mass spectrum of metabolite M6 or N8 (t_R = 14.46 min; M^+ = 286).

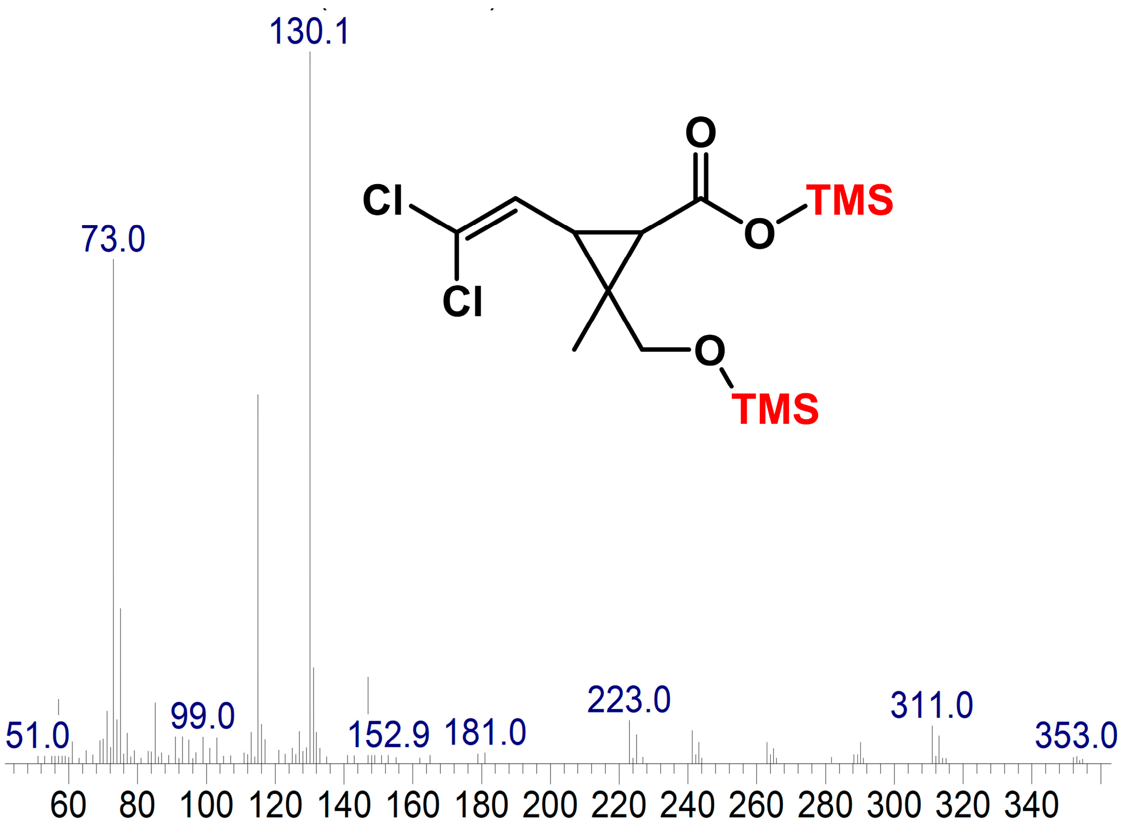

Figure S8: Mass spectrum of metabolite M7 (t_R = 14.69 min; M^+ = 368 (353+15)).

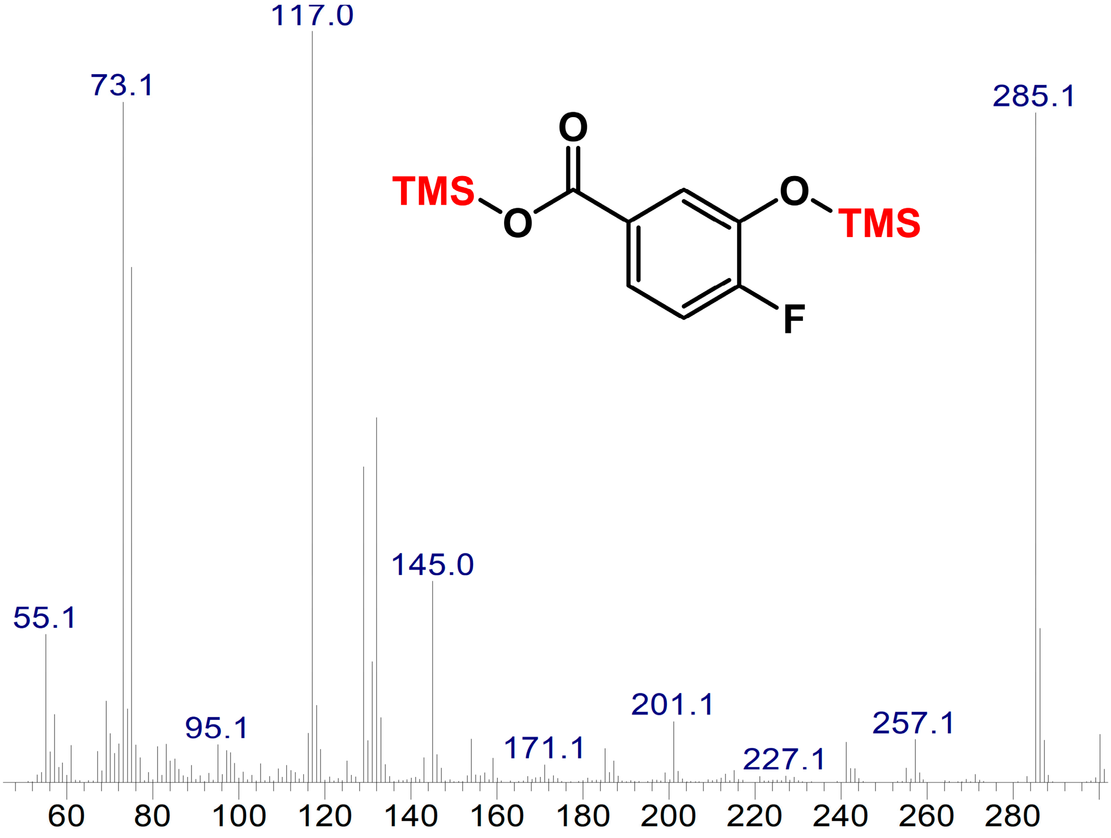

Figure S9: Mass spectrum of metabolite M8 (t_R = 15.14 min; M^+ = 300).

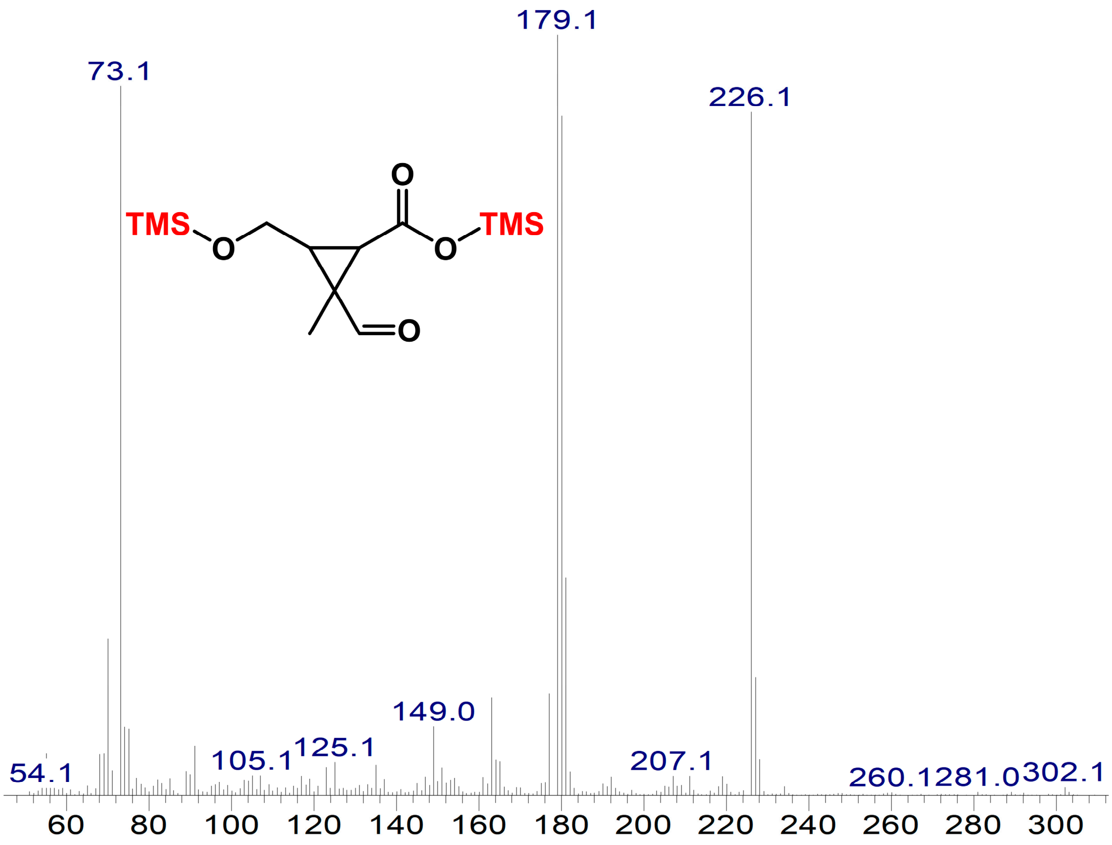

Figure S10: Mass spectrum of metabolite M9 or N9 (t_R = 15.59 min; M^+ = 302).

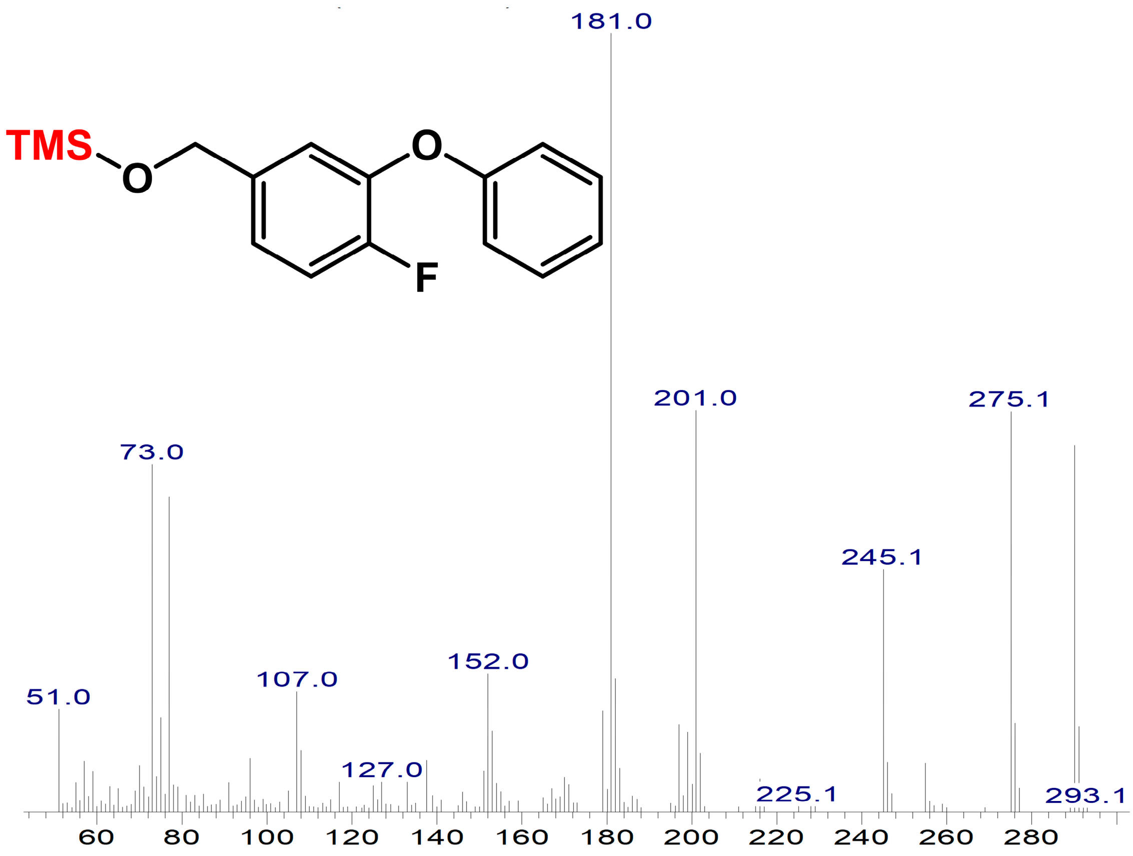

Figure S11: Mass spectrum of metabolite M10 (t_R = 15.85 min; M^+ = 290).

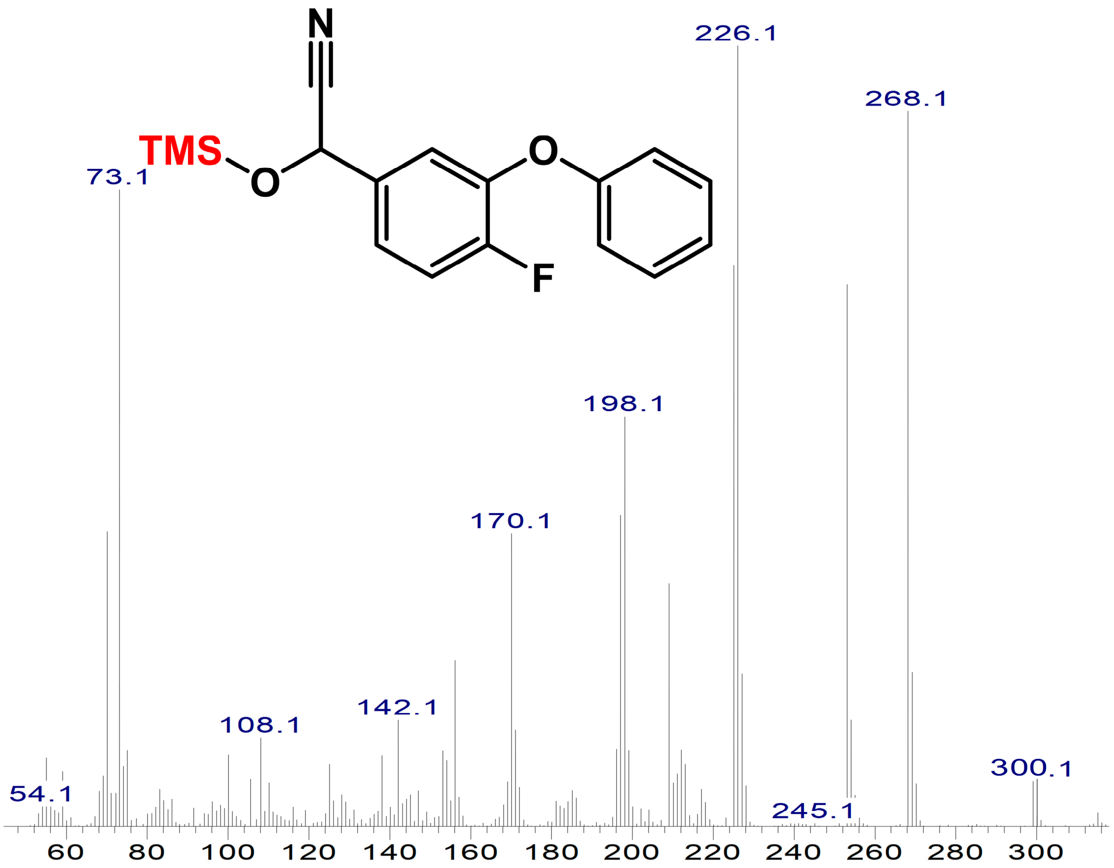

Figure S12: Mass spectrum of metabolite M11 (t_R = 16.19 min; M^+ = 315).

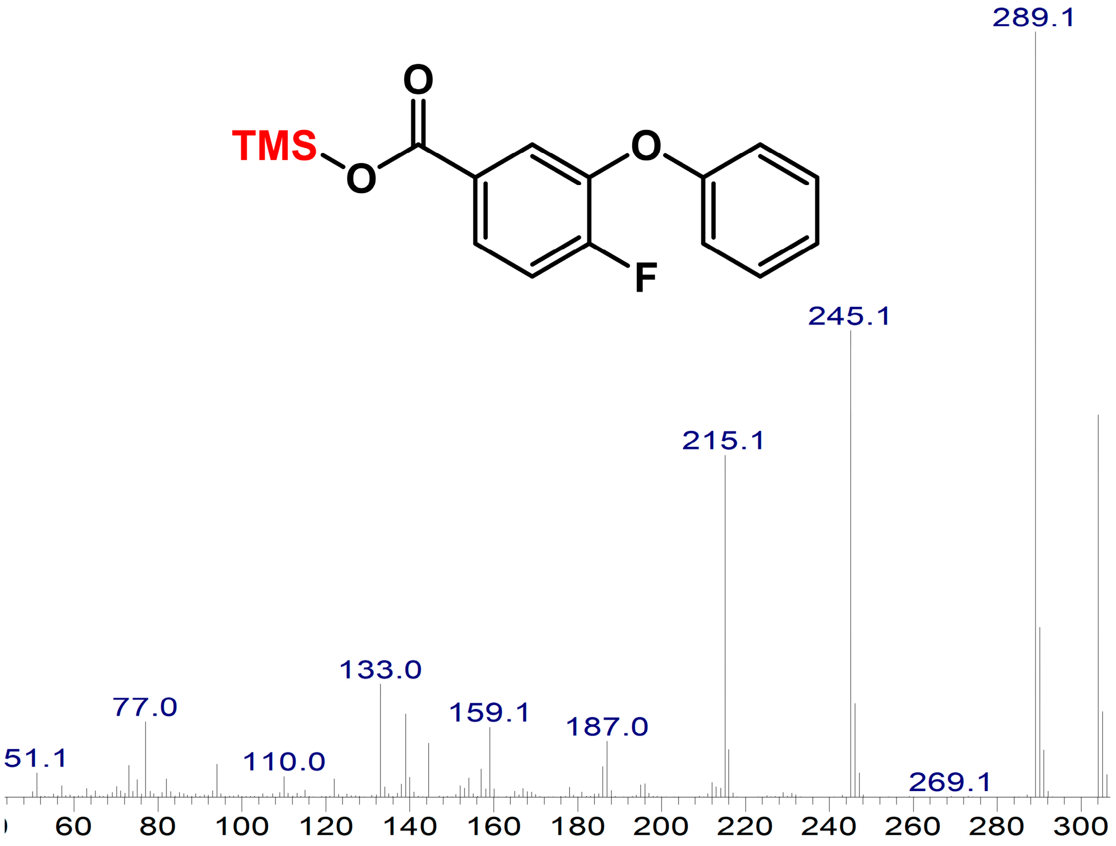

Figure S13: Mass spectrum of metabolite M12 (t_R = 16.59 min; M^+ = 304).

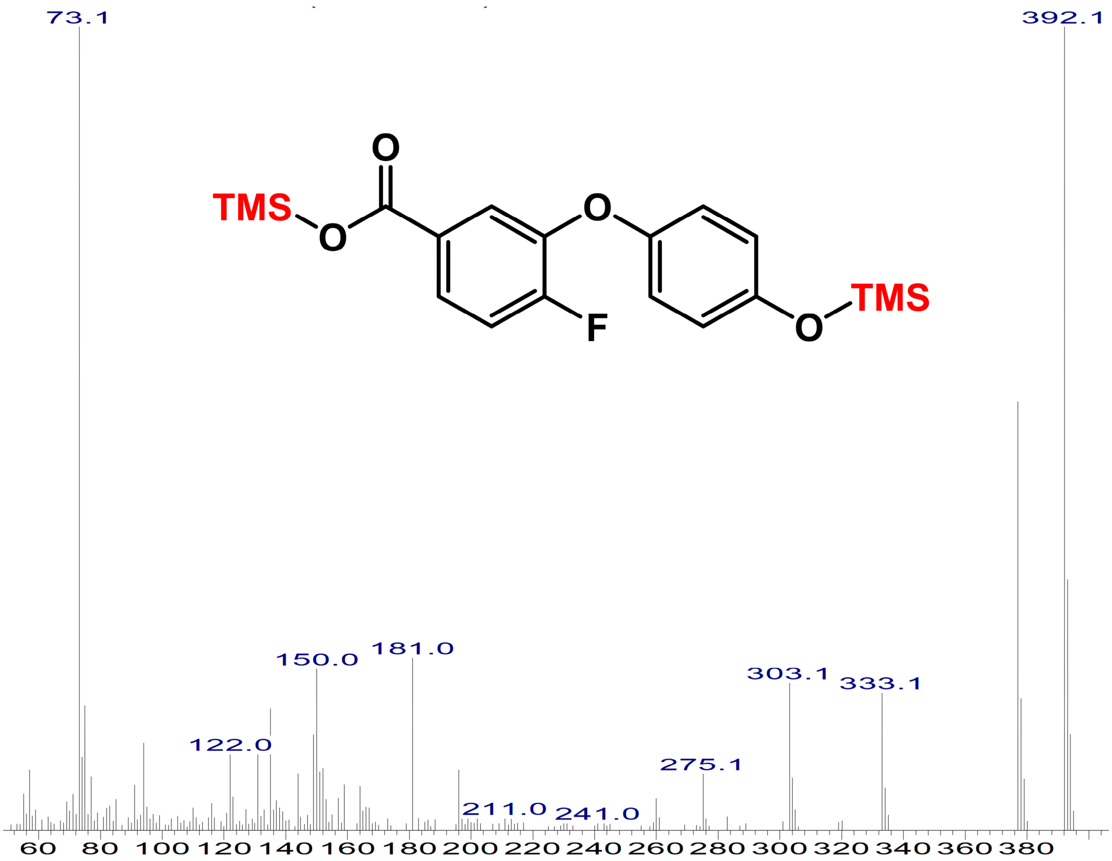

Figure S14: Mass spectrum of metabolite M13 (t_R = 19.66 min; M^+ = 392).

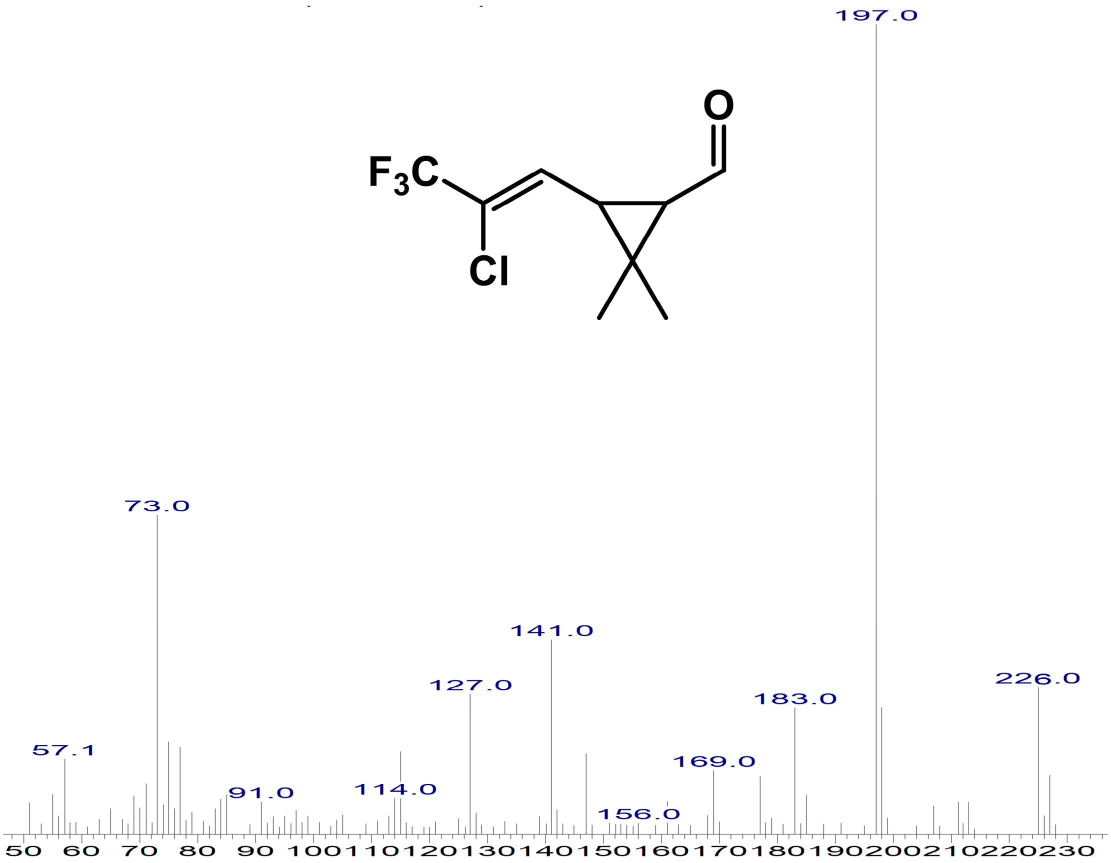

Figure S15: Mass spectrum of metabolite N1 (t_R = 8.33 min; M^+ = 226).

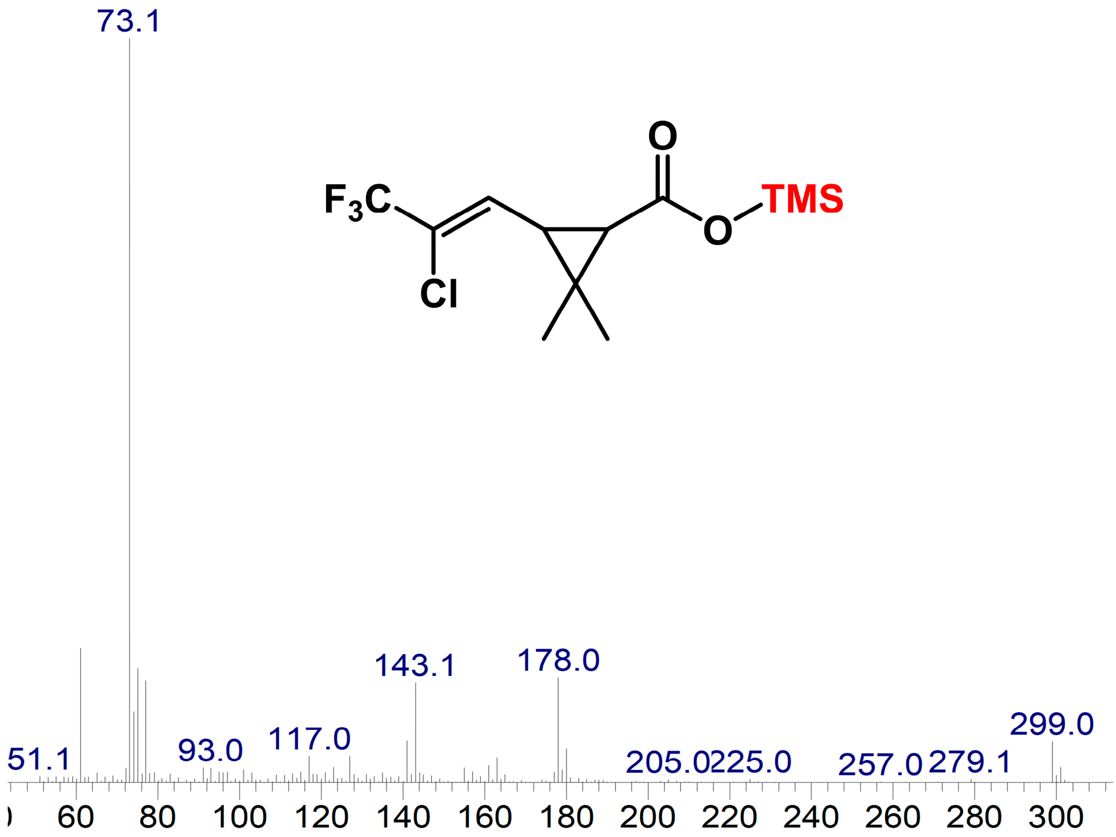

Figure S16: Mass spectrum of metabolite N2 (t_R = 9.15 min; M^+ = 314 (299+15)).

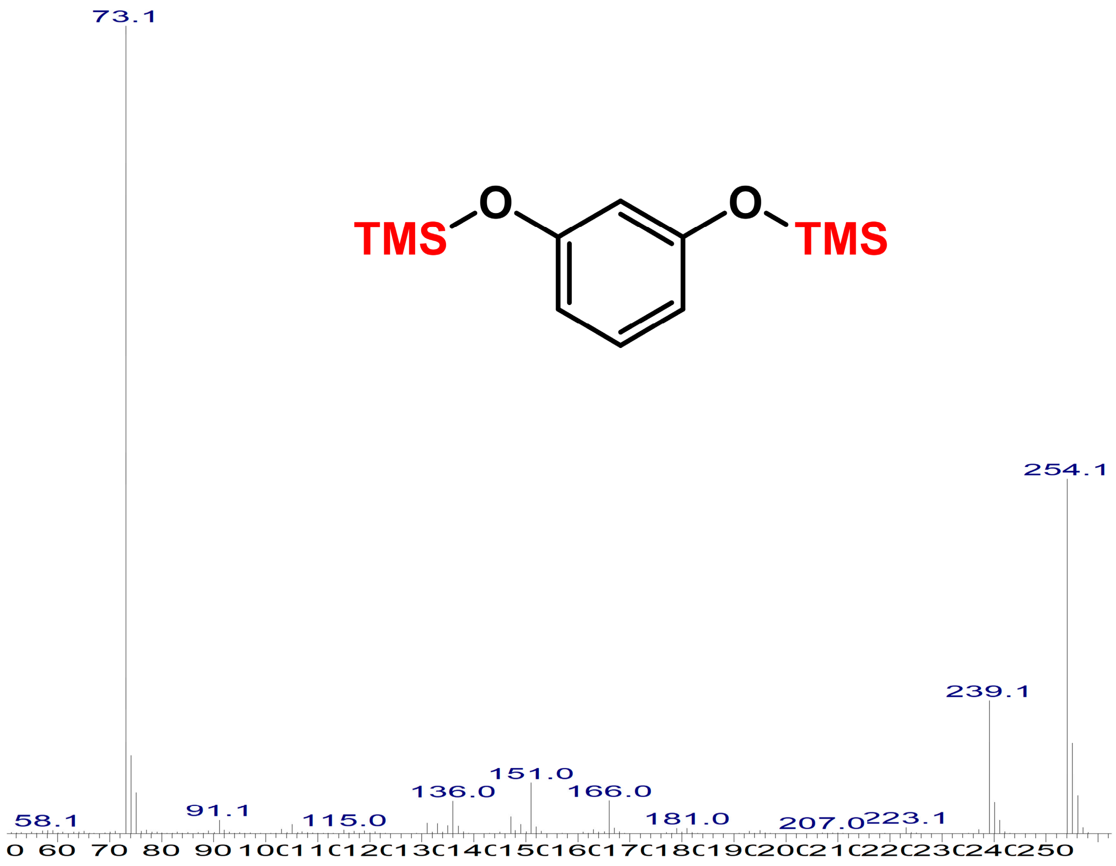

Figure S17: Mass spectrum of metabolite N4 (t_R = 10.50 min; M^+ = 254).

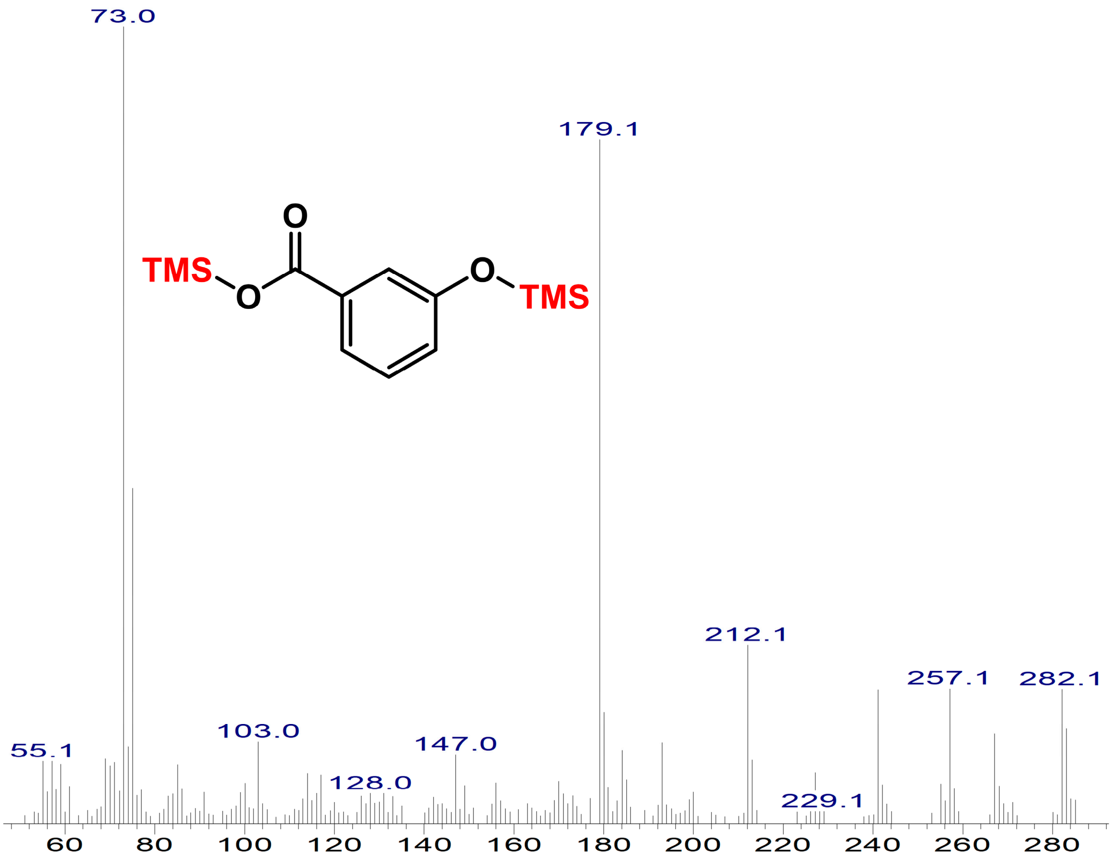

Figure S18: Mass spectrum of metabolite N5 (t_R = 12.83 min; M^+ = 282).

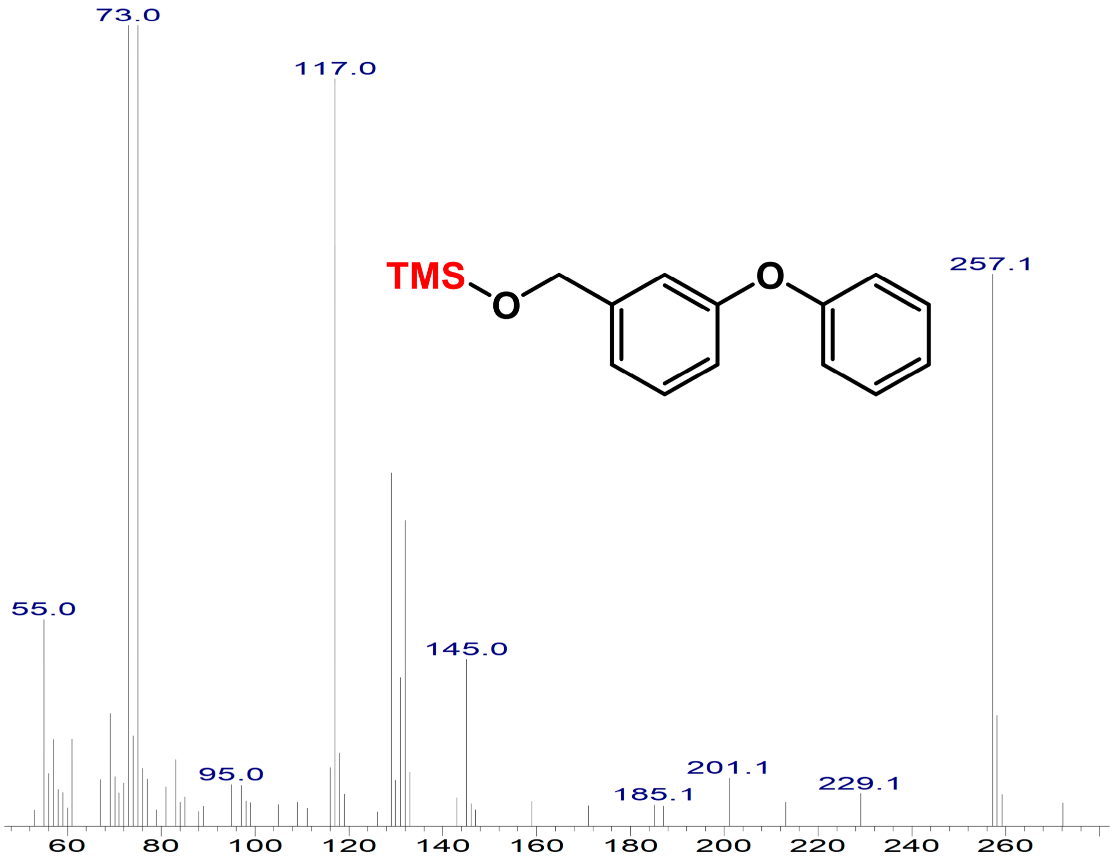

Figure S19: Mass spectrum of metabolite N7 (t_R = 13.53 min; M^+ = 272).

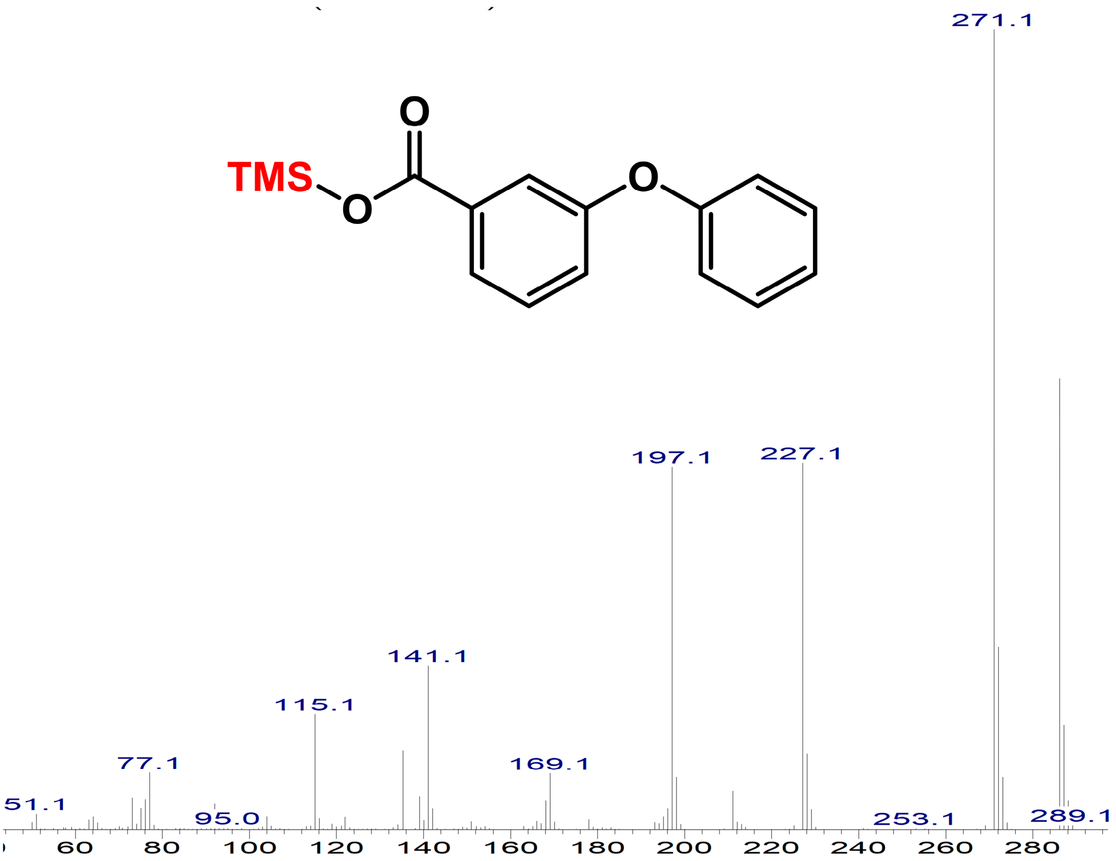

Figure S20: Mass spectrum of metabolite N10 (t_R = 16.88 min; M^+ = 286).

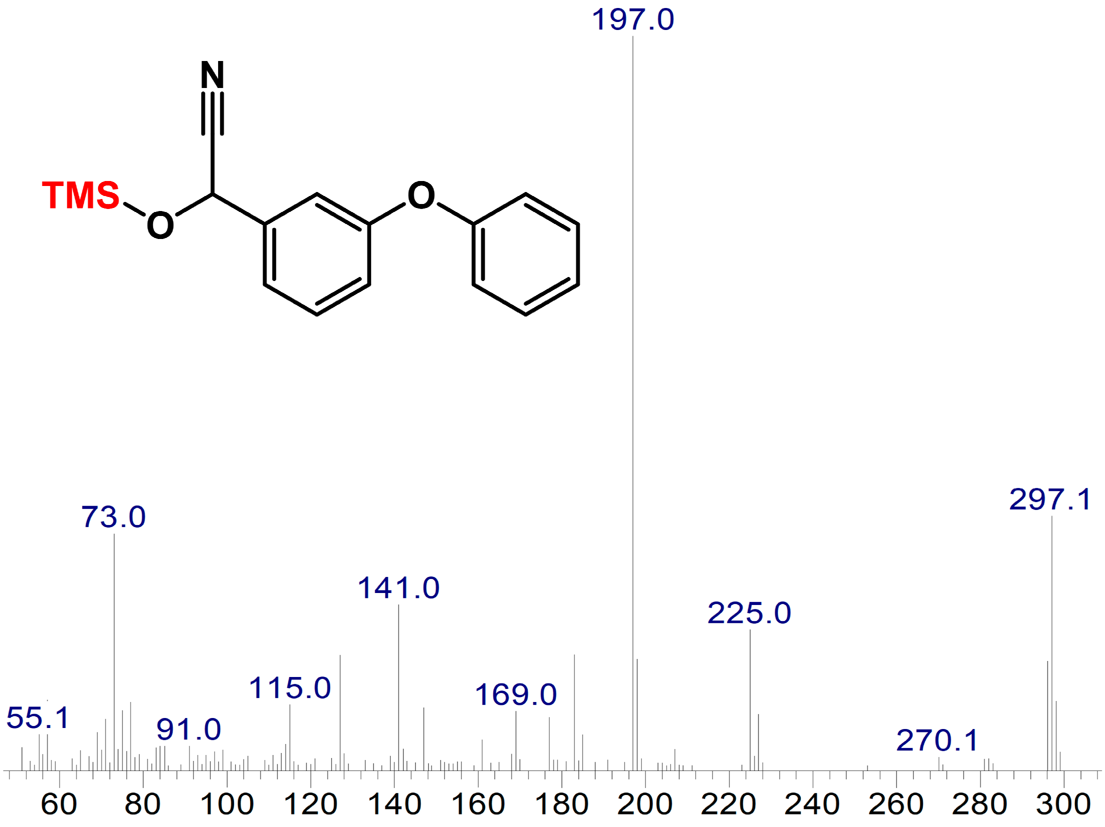

Figure S21: Mass spectrum of metabolite N11 (t_R = 18.88 min; M^+ = 297).

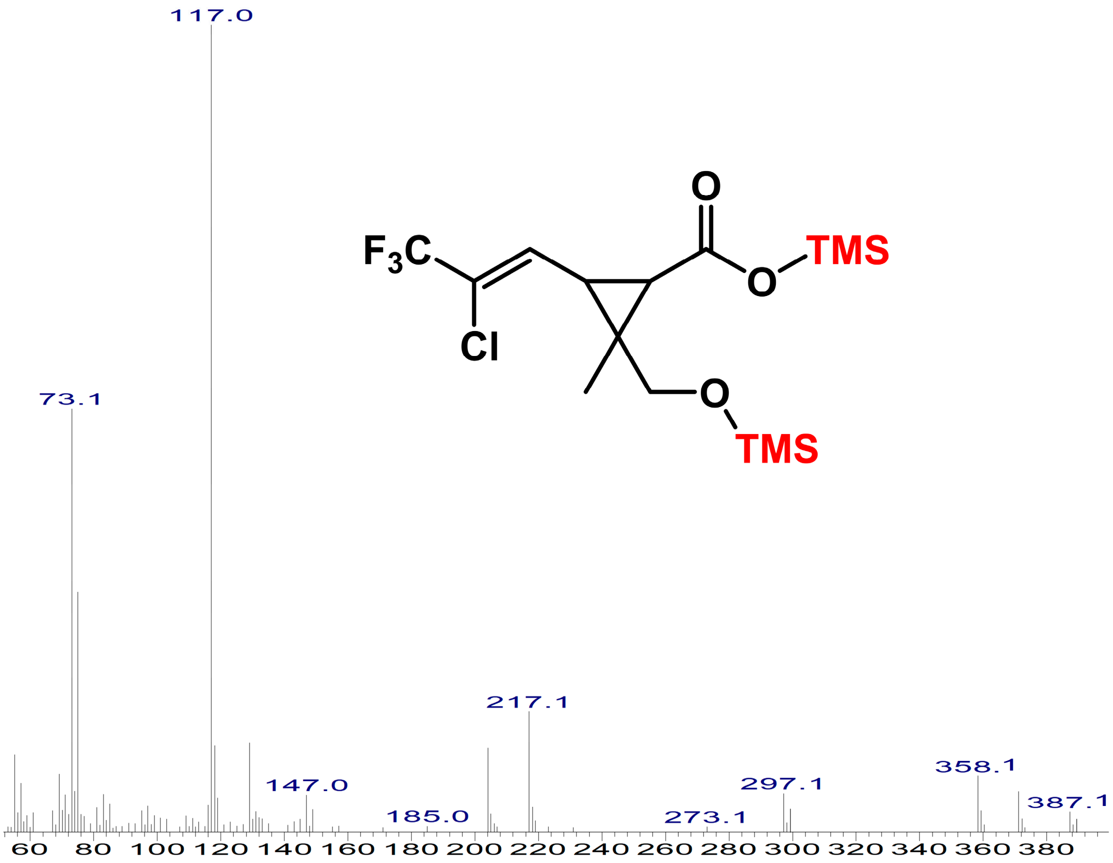

Figure S22: Mass spectrum of metabolite N12 (t_R = 19.03 min; M^+ = 402 (387+15)).

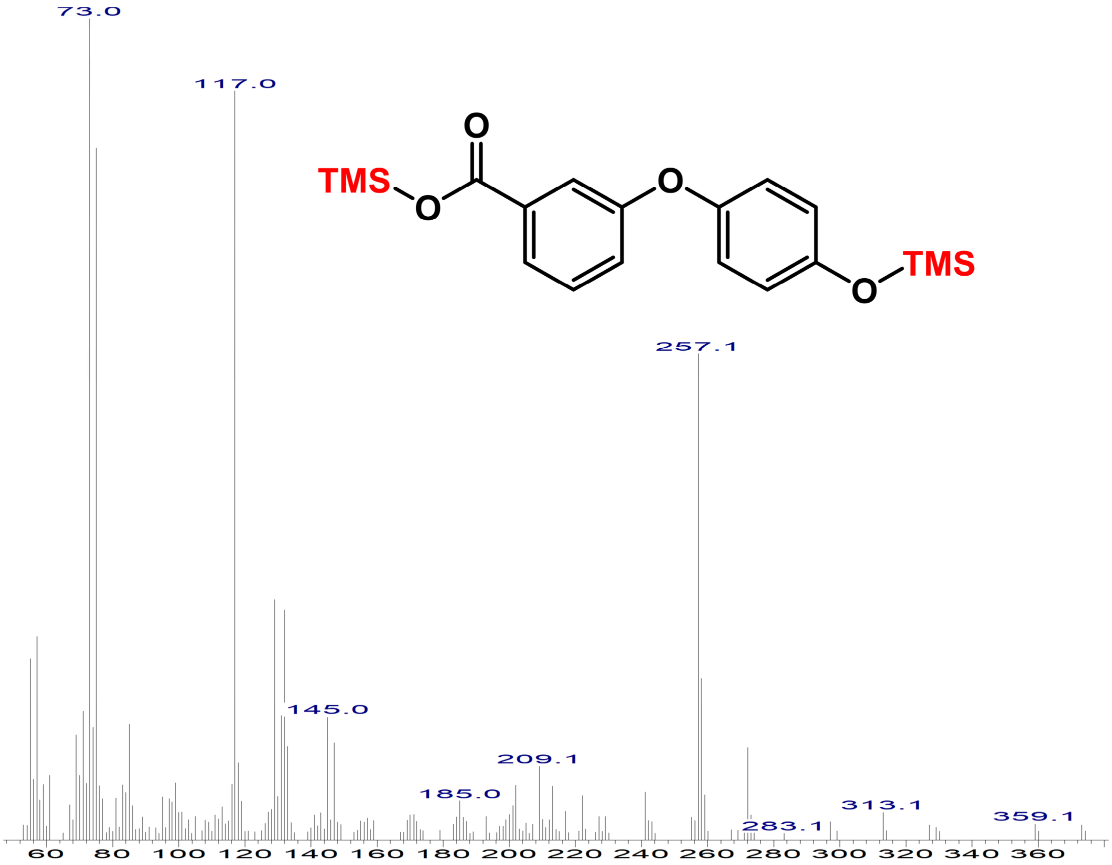

Figure S23: Mass spectrum of metabolite N13 (t_R = 20.70 min; M^+ = 374).

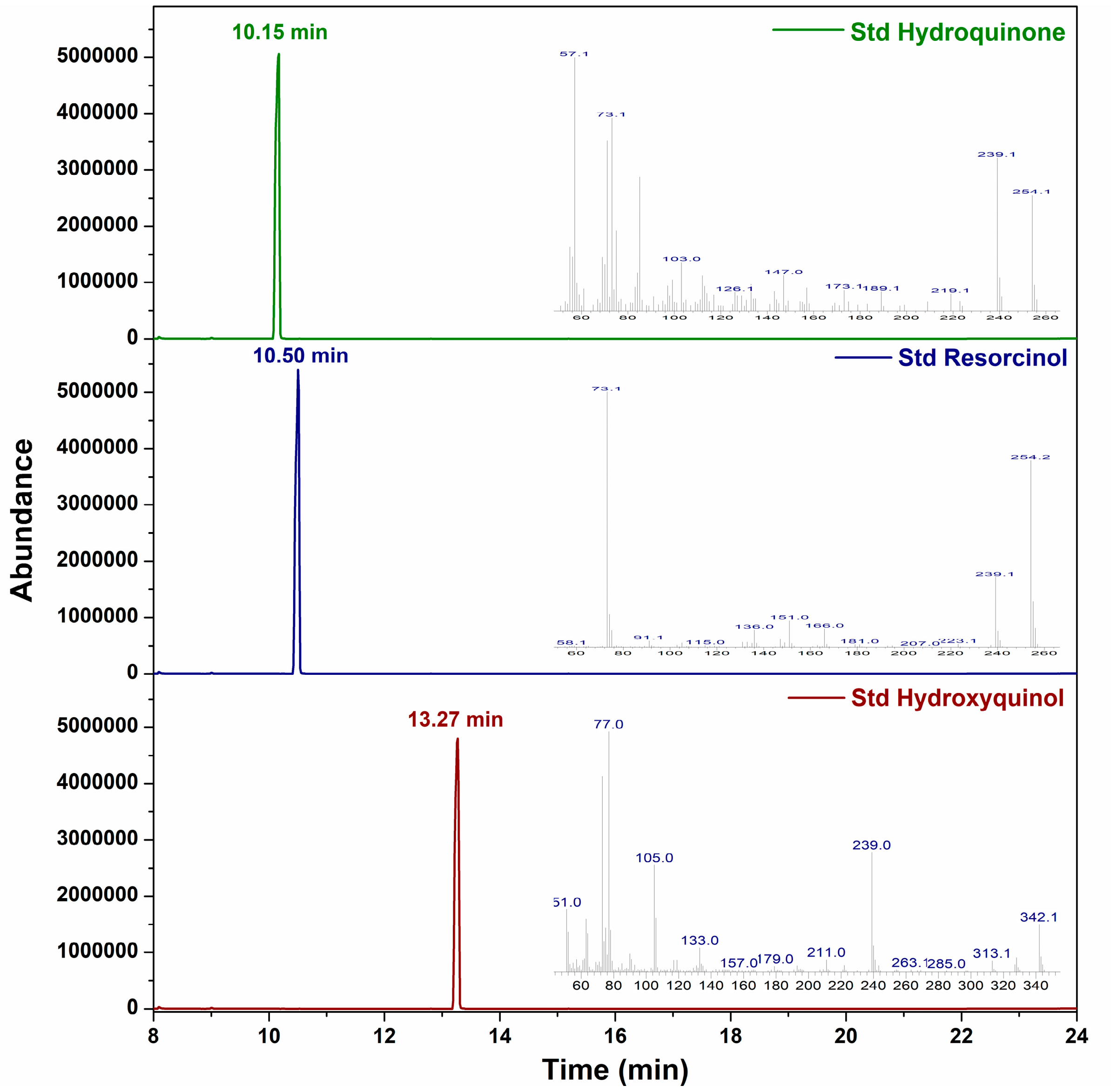

Figure S24: GC-MS analysis of standards: (A) resorcinol, (B) hydroquinone, and (C) hydroxyquinol.

.

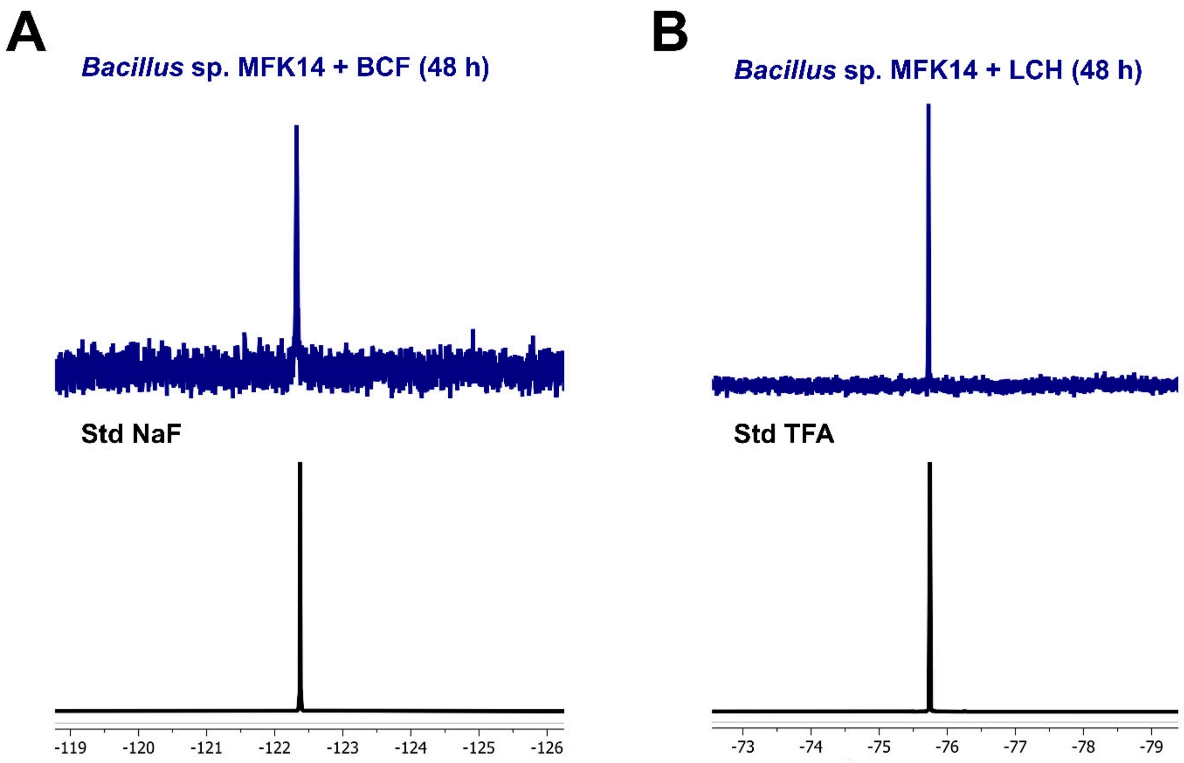

Figure S25: ^{19}F-NMR analysis confirmed the presence of (A) fluoride ion in β-cyfluthrin; and (B) trifluoroacetic acid in λ-cyhalothrin after 48 h incubation with *Bacillus* sp. MFK14.

Table S1: Putative CYP sequences were extracted from the newly sequenced genome of *Bacillus* sp. MFK14

CYP	Putative CYP gene sequence in *Bacillus* sp. MFK14
**CYP102A**	>lcl|Query_7041597:c147497-146061<br>EDGE_1897736_length_305390_cov_67.861510':EDGE_1896746_length_255_cov_302.070312'<br>ATGAAGGAAACAAGCCCGATTCCTCAACCGAAGACGTTTGGGCCGCTCGGCAATTTGCCTTTAATCGATA<br>AAGACAAGCCGACGCTTTCGCTGATCAAACTGGCGGAAGAACAGGGCCCGATTTTTCAAATCCATACACC<br>CGCGGGCACGACCATTGTAGTGTCCGGCCACGAATTGGTGAAAGAGGTTTGTGATGAAGAACGGTTTGAT<br>AAAAGCATTGAAGGCGCCTTGGAAAAGGTTCGCGCATTTTCCGGTGACGGATTGTTTACGAGCTGGACGC<br>ATGAGCCTAACTGGAGAAAAGCGCACAACATTTTGATGCCGACGTTCAGCCAGCGGGCCATGAAGGACTA<br>TCATGAGAAAATGGTCGATATCGCTGTTCAGCTCATTCAAAAATGGGCAAGGCTCAACCCGAATGAAGCA<br>GTCGATGTCCCGGGAGATATGACCCGGCTGACGCTCGACACCATTGGGCTATGCGGGTTTAACTACCGCT<br>TTAACAGTTACTACAGAGAAACGCCCCACCCGTTTATCAACAGCATGGTGCGGGCGCTTGATGAAGCGAT<br>GCATCAAATGCAGCGGCTTGATGTTCAAGATAAGCTTATGGTCAGAACAAAGCGGCAATTCCACCATGAT<br>ATTCAAACGATGTTTTCGTTAGTCGACAGCATTATTGCAGAGCGCAGATCGAATGGAGACCAGGATGAAA<br>AAGATTTGCTCGCCCGCATGCTGAATGTGGAAGATCCGGAAACGGGTGAAAAGCTCGACGACGAAAATAT<br>CCGCTTTCAAATTATTACGTTTTTGATTGCCGGCCATGAAACAACGAGTGGCCTGCTTTCCTTTGCGACT<br>TATTTTTTATTGAAGCATCCTGACAAACTGAAAAAGGCGTATGAAGAGGTCGATCGGGTGCTGACAGATG<br>CAGCGCCGACCTATAAACAAGTGCTGGAGCTTACATACATACGGATGATTTTAAATGAATCACTGCGCTT<br>ATGGCCGACAGCTCCGGCTTTCAGCCTTTATCCAAAAGAAGACACAGTCATTGGCGGGAAATTTCCGATC<br>ACGACGAATGACAGAATTTCTGTGCTGATTCCGCAGCTTCATCGTGATCGAGACGCTTGGGGAAAGGACG<br>CAGAAGAATTCCGGCCGGAACGGTTTGAGCATCAGGACCAAGTGCCTCATCATGCGTACAAACCATTCGG<br>AAATGGACAACGGGCCTGTATCGGCATGCAGTTTGCCCTTCATGAAGCCACACTTGTGTTAGGCATGATT<br>CTAAAATATTTCACATTGATTGATCATGAGAATTATGAGCTTGATATCAAACAAACCTTAACACTTAAGC<br>CGGGCGATTTTCACATCAGGGTTCAAAGCCGTAATCAGGATGCCATTCATGCAGACGTCCAGGCAGCTGA<br>AAAAGCCGCGTCTGATGAGCAAAAGGAGAAAACGGAA
**CYP106A**	>lcl|Query_170353:c595466-594396<br>EDGE_1897942_length_936390_cov_52.401534:EDGE_1888790_length_258_cov_119.633588<br>AAAAACAAACAGGATGCGTATCATCCGTTTCCATGGTACGAATCGATGAGAAAGGATTCGCCTGTTTCCT<br>TTGATGAAGAAAACCAAGTGTGGAGCGTTTTTCTTTATGATGATGTCAAAAAAGTTGTTGGGGATAAAGA<br>GCTGTTTTCCAGTTACATGCCGCAGCAGACAAGCTCTATTGGAAATTCCATCATTAACATGGACCCGCCG<br>AAGCATACAAAAATCCGTTCAGTCGTGAATAAAGCCTTTACTCCGCGCGTGATGAAGCAATGGGAACCGA<br>GAATTCAAGAAATCACAGATGAACTGATTCAAAAATGTCAGGGGCGCAGTGAGTTTGACCTTGTTCACGA<br>TTTTTCATACCCGCTTCCGGTTATTGTGATATCTGAGCTGCTGGGAGTGCCTTCAGCGCATATGGATCAG<br>TTTAAAGCATGGTCTGATCTTCTGGTCAGTACACCGAAGGATAAAAGTGAAGAAGCTGAAAAAGCCTTTT<br>TGGAAGAACGAGATAAGTGTGAGGAAGAACTGGCTGCGTTTTTTGCCGGCATCATAGAAGAAAAGCGAAA<br>CAAACCGGCACAGGATATTATTTCTATTTTAGTGGAAGCGGAAGAAACAGGCGAGAAGCTGTCCGGTGAA<br>GAGCTGATTCCGTTTTGCACGCTGCTGCTGGTGGCCGGAAATGAAACCACTACAAACCTGATTTCAAATG<br>CGATGTACAGCATATTAGAAACGCCAGGCGTTTACGAGGAACTGCGCAGCCATCCTGAACTGATGCCTCA<br>GGCAGTCGAGGAAGCCTTACGTTTCAGAGCGCCCGCCCCGGTTTTGAGACGCATTGCCAAGCGGGATACG<br>GAAATCGGGGGGCATCTGATTAAAGAAGGTGATATGGTTTTGGCGTTTGTGGCATCGGCAAATCGTGATG<br>AAGCGAAGTTTGACAGGCCGCACATGTTTGATATCCACCGCCATCCCAATCCGCATATTGCGTTTGGTCA<br>TGGCATTCATTTTTGCCTTGGGGCTCCGCTTGCCCGTCTTGAAGCAAATATCGCGTTAACGTCTTTGATT<br>TCTGCTTTTCCTCAAATGGAG
**CYP106B**	>lcl|Query_5253279:c595466-594378<br>EDGE_1897942_length_936390_cov_52.401534:EDGE_1888790_length_258_cov_119.633588<br>AAAAACAAACAGGATGCGTATCATCCGTTTCCATGGTACGAATCGATGAGAAAGGATTCGCCTGTTTCCT<br>TTGATGAAGAAAACCAAGTGTGGAGCGTTTTTCTTTATGATGATGTCAAAAAAGTTGTTGGGGATAAAGA<br>GCTGTTTTCCAGTTACATGCCGCAGCAGACAAGCTCTATTGGAAATTCCATCATTAACATGGACCCGCCG<br>AAGCATACAAAAATCCGTTCAGTCGTGAATAAAGCCTTTACTCCGCGCGTGATGAAGCAATGGGAACCGA<br>GAATTCAAGAAATCACAGATGAACTGATTCAAAAATGTCAGGGGCGCAGTGAGTTTGACCTTGTTCACGA<br>TTTTTCATACCCGCTTCCGGTTATTGTGATATCTGAGCTGCTGGGAGTGCCTTCAGCGCATATGGATCAG<br>TTTAAAGCATGGTCTGATCTTCTGGTCAGTACACCGAAGGATAAAAGTGAAGAAGCTGAAAAAGCCTTTT<br>TGGAAGAACGAGATAAGTGTGAGGAAGAACTGGCTGCGTTTTTTGCCGGCATCATAGAAGAAAAGCGAAA<br>CAAACCGGCACAGGATATTATTTCTATTTTAGTGGAAGCGGAAGAAACAGGCGAGAAGCTGTCCGGTGAA<br>GAGCTGATTCCGTTTTGCACGCTGCTGCTGGTGGCCGGAAATGAAACCACTACAAACCTGATTTCAAATG<br>CGATGTACAGCATATTAGAAACGCCAGGCGTTTACGAGGAACTGCGCAGCCATCCTGAACTGATGCCTCA<br>GGCAGTCGAGGAAGCCTTACGTTTCAGAGCGCCCGCCCCGGTTTTGAGACGCATTGCCAAGCGGGATACG<br>GAAATCGGGGGGCATCTGATTAAAGAAGGTGATATGGTTTTGGCGTTTGTGGCATCGGCAAATCGTGATG<br>AAGCGAAGTTTGACAGGCCGCACATGTTTGATATCCACCGCCATCCCAATCCGCATATTGCGTTTGGTCA<br>TGGCATTCATTTTTGCCTTGGGGCTCCGCTTGCCCGTCTTGAAGCAAATATCGCGTTAACGTCTTTGATT

	TCTGCTTTTCCTCAAATGGAGTGCGTCAGTATCACTCCG
**CYP107DY**	>lcl|Query_488221:29128-30285<br>EDGE_1897942_length_936390_cov_52.401534:EDGE_1888790_length_258_cov_119.633588<br>AATCCTTTTTCAGTTTTAGGACGATTTAGAGAGGAAGAGCCCATTCACCGATTTGAATTAAAACGGTTCG<br>GAGCCACATATCCGGCCTGGTTAATTACCCGATACGATGATTGTATGGCCTTTTTAAAAGACAATCGAAT<br>TACAAGAGACGTAAAAAATGTGATGAACCAAGAACAAATCAAAATGCTCAACGTTAGTGAAGATATCGAT<br>TTTGTATCCGATCATATGCTGGCAAAAGACACACCTGACCATACCCGCCTGAGATCACTTGTTCATCAAG<br>CATTTACTCCCCGAACCATTGAAAATCTGCGCGGCAGCATTGAACAAATTGCTGAACAGCTTTTAGATGA<br>AATGGAAAAAGAAAATAAAGCGGATATCATGAAATCCTTCGCTTCCCCTTTGCCTTTTATTGTTATATCT<br>GAATTGATGGGAATCCCAAAAGAAGATCGGTCACAGTTTCAAATCTGGACCAATGCGATGGTTGATACCT<br>CTGAAGGTAATAGAGAGCTGACAAATCAGGCCCTTCGTGAATTTAAAGATTATATCGCTAAGCTGATCCA<br>TGACAGAAGAATAAAGCCAAAAGACGATTTAATCAGCAAACTTGTGCATGCTGAGGAAAACGGCAGCAAG<br>TTAAGCGAAAAAGAGCTCTATTCGATGCTGTTCTTGCTCGTTGTAGCCGGCCTTGAGACGACTGTTAACT<br>TACTCGGCTCAGGCACACTCGCATTGCTGCAGCACAAGAAGGAATGTGAGAAGCTCAAGCAGTATCCTGA<br>AATGATCGCTACAGCGGTTGAAGAATTGCTGCGATACACCTCACCTGTCGTTATGATGGCAAATCGGTGG<br>GCCATCGAAGACTTTACATATAAGGGGCATTCGATCAAAAGAGGAGACATGATTTTTATAGGCATCGTAT<br>CTGCCAATCGCGACCCGAATTTTTTCGAGAACCCCGAAATATTAAATATAAACCGGTCGCCTAACAGACA<br>TATTTCTTTCGGTTTTGGCATTCATTTCTGCTTAGGAGCGCCTCTTGCCAGGCTGGAAGGCCACATTGCA<br>TTTAACGCACTTTTGAAGAGATTTCCTGATATTCAACTTGCGGTTGCACCTGATGACATTCAATGGAGAA<br>AAAATGTCTTTTTAAGAGGATTAGAAAGTCTCCCTGTT
**CYP107H**	>lcl|Query_7145494:c243212-242028<br>EDGE_1897868_length_325345_cov_56.588024:EDGE_1888790_length_258_cov_119.633588<br>GTGACAATTGCATCGTCAACTGCATCTTATGAGTTTTTGAAAAACCCATATTCTTTTTACGACACATTGC<br>GAGCTGTTCATCCTATCTATAAAGGGAGTTTCTTAAAATATCCGGGCTGGTATGTCACAGGATATGAAGA<br>AACGGCTGCTATTTTGAAAGATGCGAGATTCAAAGTCCGCACCCCGCTGCCTGAGAGCTCAACCAAATAT<br>CAGGACCTTTCACATGTGCAAAATCAAATGATGCTGTTTCAGAACCGGCCTGATCATAGACGATTGCGGA<br>CGCTTGCCAGCGGAGCGTTTACGCCGAGAACGACAGAGAGTTATCAGCCGTATATCATTGAAACTGTCCA<br>TCATTTGCTTGATCAAGTGCAAGGTAAAAAAAAGATGGAGGTCATTTCGGACTTTGCTTTTCCTTTAGCA<br>AGTTTTGTCATAGCTAACATTATAGGTGTACCGGAGGAAGATAGGGAGCAATTAAAGGAGTGGGCTGCGA<br>GTCTCATTCAAACGATTGATTTTACCCGCTCAAGAAAGGCATTAACAGAGGGCAATATTATGGCTGTGCA<br>GGCTATGGCATATTTCAAAGAGCTGATTCAAAAGAGAAAACGCCACCCTCAACAGGATATGATCAGCATG<br>CTCTTGAAGGGGAGAGAAAAGGATAAGCTGACGGAAGAGGAGGCGGCATCTACGTGCATATTGCTGGCGA<br>TCGCCGGACATGAGACAACGGTCAATCTCATCAGCAATTCAGTCCTTTGTCTGCTGCAGCATCCAGAACA<br>GCTTTTGAAACTGAGAGAAAATCCAGATCTTATTGGTACCGCAGTCGAGGAATGTTTACGCTATGAAAGC<br>CCCACGCAAATGACAGCCAGAGTTGCGTCAGAGGATATTGACATCTGCGGGGTGACGATCCGTCAAGGAG<br>AACAAGTCTATCTTTTGTTAGGAGCGGCTAATCGAGACCCTAGCATATTCACGAACCCCGATGTCTTCGA<br>TATTACGAGAAGTCCTAATCCGCATCTTTCATTCGGGCATGGCCATCATGTTTGCTTAGGGTCCTCGCTG<br>GCACGATTAGAAGCGCAAATTGCGATTAACACTCTTCTGCAGCGAATGCCCAGCCTTAAGCTCGCGGATT<br>TTGAATGGCGGTATCGGCCGCTTTTTGGATTTCGGGCGCTTGAGGAGCTGCCGGTGACTTTTGAA
**CYP107J**	>lcl|Query_3673089:29077-30186<br>EDGE_1897942_length_936390_cov_52.401534:EDGE_1888790_length_258_cov_119.633588<br>ATGCAAATGGAAAAAATTGATGTTTCATCCGCATGGTAAAGAGTTTCATCACAATCCTTTTTCAGTTTTAG<br>GACGATTTAGAGAGGAAGAGCCCATTCACCGATTTGAATTAAAACGGTTCGGAGCCACATATCCGGCCTG<br>GTTAATTACCCGATACGATGATTGTATGGCCTTTTTAAAAGACAATCGAATTACAAGAGACGTAAAAAAT<br>GTGATGAACCAAGAACAAATCAAAATGCTCAACGTTAGTGAAGATATCGATTTTGTATCCGATCATATGC<br>TGGCAAAAGACACACCTGACCATACCCGCCTGAGATCACTTGTTCATCAAGCATTTACTCCCCGAACCAT<br>TGAAAATCTGCGCGGCAGCATTGAACAAATTGCTGAACAGCTTTTAGATGAAATGGAAAAAGAAAATAAA<br>GCGGATATCATGAAATCCTTCGCTTCCCCTTTGCCTTTTATTGTTATATCTGAATTGATGGGAATCCCAA<br>AAGAAGATCGGTCACAGTTTCAAATCTGGACCAATGCGATGGTTGATACCTCTGAAGGTAATAGAGAGCT<br>GACAAATCAGGCCCTTCGTGAATTTAAAGATTATATCGCTAAGCTGATCCATGACAGAAGAATAAAGCCA<br>AAAGACGATTTAATCAGCAAACTTGTGCATGCTGAGGAAAACGGCAGCAAGTTAAGCGAAAAAGAGCTCT<br>ATTCGATGCTGTTCTTGCTCGTTGTAGCCGGCCTTGAGACGACTGTTAACTTACTCGGCTCAGGCACACT<br>CGCATTGCTGCAGCACAAGAAGGAATGTGAGAAGCTCAAGCAGTATCCTGAAATGATCGCTACAGCGGTT<br>GAAGAATTGCTGCGATACACCTCACCTGTCGTTATGATGGCAAATCGGTGGGCCATCGAAGACTTTACAT<br>ATAAGGGGCATTCGATCAAAAGAGGAGACATGATTTTTATAGGCATCGTATCTGCCAATCGCGACCCGAA<br>TTTTTTCGAGAACCCCGAAATATTAAATATAAACCGGTCGCCTAACAGACATATTTCTTTCGGTTTTGGC<br>ATTCATTTCTGCTTAGGAGCGCCTCTTGCCAGGCTGGAAGGCCACATTGCATTTAACGCA
**CYP107K**	>lcl|Query_3673089:29077-30186<br>EDGE_1897942_length_936390_cov_52.401534:EDGE_1888790_length_258_cov_119.633588<br>ATGCAAATGGAAAAAATTGATGTTTCATCCGCATGGTAAAGAGTTTCATCACAATCCTTTTTCAGTTTTAG<br>GACGATTTAGAGAGGAAGAGCCCATTCACCGATTTGAATTAAAACGGTTCGGAGCCACATATCCGGCCTG<br>GTTAATTACCCGATACGATGATTGTATGGCCTTTTTAAAAGACAATCGAATTACAAGAGACGTAAAAAAT<br>GTGATGAACCAAGAACAAATCAAAATGCTCAACGTTAGTGAAGATATCGATTTTGTATCCGATCATATGC

	TGGCAAAAGACACACCTGACCATACCCGCCTGAGATCACTTGTTCATCAAGCATTTACTCCCCGAACCAT<br>TGAAAATCTGCGCGGCAGCATTGAACAAATTGCTGAACAGCTTTTAGATGAAATGGAAAAAGAAAATAAA<br>GCGGATATCATGAAATCCTTCGCTTCCCCTTTGCCTTTTATTGTTATATCTGAATTGATGGGAATCCCAA<br>AAGAAGATCGGTCACAGTTTCAAATCTGGACCAATGCGATGGTTGATACCTCTGAAGGTAATAGAGAGCT<br>GACAAATCAGGCCCTTCGTGAATTTAAAGATTATATCGCTAAGCTGATCCATGACAGAAGAATAAAGCCA<br>AAAGACGATTTAATCAGCAAACTTGTGCATGCTGAGGAAAACGGCAGCAAGTTAAGCGAAAAAGAGCTCT<br>ATTCGATGCTGTTCTTGCTCGTTGTAGCCGGCCTTGAGACGACTGTTAACTTACTCGGCTCAGGCACACT<br>CGCATTGCTGCAGCACAAGAAGGAATGTGAGAAGCTCAAGCAGTATCCTGAAATGATCGCTACAGCGGTT<br>GAAGAATTGCTGCGATACACCTCACCTGTCGTTATGATGGCAAATCGGTGGGCCATCGAAGACTTTACAT<br>ATAAGGGGCATTCGATCAAAAGAGGAGACATGATTTTTATAGGCATCGTATCTGCCAATCGCGACCCGAA<br>TTTTTTCGAGAACCCCGAAATATTAAATATAAACCGGTCGCCTAACAGACATATTTCTTTCGGTTTTGGC<br>ATTCATTTCTGCTTAGGAGCGCCTCTTGCCAGGCTGGAAGGCCACATTGCATTTAACGCA
**CYP109A**	>lcl|Query_7184441:c595463-594330<br>EDGE_1897942_length_936390_cov_52.401534:EDGE_1888790_length_258_cov_119.633588<br>AACAAACAGGATGCGTATCATCCGTTTCCATGGTACGAATCGATGAGAAAGGATTCGCCTGTTTCCTTTG<br>ATGAAGAAAACCAAGTGTGGAGCGTTTTTCTTTATGATGATGTCAAAAAAGTTGTTGGGGATAAAGAGCT<br>GTTTTCCAGTTACATGCCGCAGCAGACAAGCTCTATTGGAAATTCCATCATTAACATGGACCCGCCGAAG<br>CATACAAAAATCCGTTCAGTCGTGAATAAAGCCTTTACTCCGCGCGTGATGAAGCAATGGGAACCGAGAA<br>TTCAAGAAATCACAGATGAACTGATTCAAAAATGTCAGGGGCGCAGTGAGTTTGACCTTGTTCACGATTT<br>TTCATACCCGCTTCCGGTTATTGTGATATCTGAGCTGCTGGGAGTGCCTTCAGCGCATATGGATCAGTTT<br>AAAGCATGGTCTGATCTTCTGGTCAGTACACCGAAGGATAAAAGTGAAGAAGCTGAAAAAGCCTTTTTGG<br>AAGAACGAGATAAGTGTGAGGAAGAACTGGCTGCGTTTTTTGCCGGCATCATAGAAGAAAAGCGAAACAA<br>ACCGGCACAGGATATTATTTCTATTTTAGTGGAAGCGGAAGAAACAGGCGAGAAGCTGTCCGGTGAAGAG<br>CTGATTCCGTTTTGCACGCTGCTGCTGGTGGCCGGAAATGAAACCACTACAAACCTGATTTCAAATGCGA<br>TGTACAGCATATTAGAAACGCCAGGCGTTTACGAGGAACTGCGCAGCCATCCTGAACTGATGCCTCAGGC<br>AGTCGAGGAAGCCTTACGTTTCAGAGCGCCCGCCCCGGTTTTGAGACGCATTGCCAAGCGGGATACGGAA<br>ATCGGGGGGCATCTGATTAAAGAAGGTGATATGGTTTTGGCGTTTGTGGCATCGGCAAATCGTGATGAAG<br>CGAAGTTTGACAGGCCGCACATGTTTGATATCCACCGCCATCCCAATCCGCATATTGCGTTTGGTCATGG<br>CATTCATTTTTGCCTTGGGGCTCCGCTTGCCCGTCTTGAAGCAAATATCGCGTTAACGTCTTTGATTTCT<br>GCTTTTCCTCAAATGGAGTGCGTCAGTATCACTCCGATTGAAAACAGTGTGATATACGGATTAAAGAGCT<br>TCCGTGTGAAAATG
**CYP109B**	>lcl|Query_7215909:c595469-594330<br>EDGE_1897942_length_936390_cov_52.401534:EDGE_1888790_length_258_cov_119.633588<br>GGGAAAAACAAACAGGATGCGTATCATCCGTTTCCATGGTACGAATCGATGAGAAAGGATTCGCCTGTTT<br>CCTTTGATGAAGAAAACCAAGTGTGGAGCGTTTTTCTTTATGATGATGTCAAAAAAGTTGTTGGGGATAA<br>AGAGCTGTTTTCCAGTTACATGCCGCAGCAGACAAGCTCTATTGGAAATTCCATCATTAACATGGACCCG<br>CCGAAGCATACAAAAATCCGTTCAGTCGTGAATAAAGCCTTTACTCCGCGCGTGATGAAGCAATGGGAAC<br>CGAGAATTCAAGAAATCACAGATGAACTGATTCAAAAATGTCAGGGGCGCAGTGAGTTTGACCTTGTTCA<br>CGATTTTTCATACCCGCTTCCGGTTATTGTGATATCTGAGCTGCTGGGAGTGCCTTCAGCGCATATGGAT<br>CAGTTTAAAGCATGGTCTGATCTTCTGGTCAGTACACCGAAGGATAAAAGTGAAGAAGCTGAAAAAGCCT<br>TTTTGGAAGAACGAGATAAGTGTGAGGAAGAACTGGCTGCGTTTTTTGCCGGCATCATAGAAGAAAAGCG<br>AAACAAACCGGCACAGGATATTATTTCTATTTTAGTGGAAGCGGAAGAAACAGGCGAGAAGCTGTCCGGT<br>GAAGAGCTGATTCCGTTTTGCACGCTGCTGCTGGTGGCCGGAAATGAAACCACTACAAACCTGATTTCAA<br>ATGCGATGTACAGCATATTAGAAACGCCAGGCGTTTACGAGGAACTGCGCAGCCATCCTGAACTGATGCC<br>TCAGGCAGTCGAGGAAGCCTTACGTTTCAGAGCGCCCGCCCCGGTTTTGAGACGCATTGCCAAGCGGGAT<br>ACGGAAATCGGGGGGCATCTGATTAAAGAAGGTGATATGGTTTTGGCGTTTGTGGCATCGGCAAATCGTG<br>ATGAAGCGAAGTTTGACAGGCCGCACATGTTTGATATCCACCGCCATCCCAATCCGCATATTGCGTTTGG<br>TCATGGCATTCATTTTTGCCTTGGGGCTCCGCTTGCCCGTCTTGAAGCAAATATCGCGTTAACGTCTTTG<br>ATTTCTGCTTTTCCTCAAATGGAGTGCGTCAGTATCACTCCGATTGAAAACAGTGTGATATACGGATTAA<br>AGAGCTTCCGTGTGAAAATG
**CYP134A**	>lcl|Query_7233442:266746-267960<br>EDGE_1896638_length_404824_cov_85.685063':EDGE_1775944_length_246_cov_51.352941',EDGE_185<br>9348_length_6187_cov_9.470462'<br>ATGAGCCAATCGATTAAATTGTTTAGTGTGCTTTCTGATCAATTTCAAAACAATCCATATGCTTATTTTT<br>CACAACTGCGGGAGGAAGATCCGGTTCATTATGAAGAGTCGATAGACAGTTATTTTATCAGCCGCTATCA<br>TGATGTCCGCTATATCCTTCAGCATCCGGATATCTTCACGACGAAATCACTTGTTGAGCGTGCCGAACCA<br>GTCATGCGAGGCCCTGTGCTGGCCCAAATGCATGGAAAAGAACACTCTGCCAAAAGAAGAATTGTAGTGA<br>GAAGCTTTATCGGTGACGCACTGGATCATCTGTCTCCATTGATTAAACAAAATGCAGAAAACTTGTTAGC<br>GCCTTATCTTGAAAGAGGGAAAATTGATCTCGTCAATGATTTTGGAAAGACGTTTGCGGTGTGCGTCACG<br>ATGGACATGCTCGGGCTGGATAAAAGAGACCATGAAAAAATCTCTGAGTGGCACAGCGGAGTTGCCGATT<br>TTATCACGAGTATCTCTCAATCTCCTGAAGCGCGGGCACATTCGTTATGGTGCAGCGAACAGCTTTCCCA<br>ATACTTGATGCCGGTCATTAAAGAACGTCGCGTCAATCCGGGATCAGATTTAATTTCGATCCTATGTACT<br>TCTGAATATGAAGGCATGGCGCTGTCGGACAAGGATATACTCGCACTGATTCTTAATGTGCTGTTAGCCG

	CAACGGAACCGGCTGATAAGACGCTGGCACTGATGATCTACCATTTGCTCAACAATCCTGAGCAGATGAA<br>TGATGTTTTGGCTGACCGTTCGTTAGTTCCGAGAGCCATTGCGGAGACATTGCGTTATAAACCGCCGGTT<br>CAGCTGATTCCGCGGCAGCTGTCCCAAGATACAGTGGTCGGCGGTATGGAAATCAAAAAAGATACGATTG<br>TTTTTTGTATGATCGGTGCGGCTAACCGGGACCCTGAAGCATTTGAACAGCCTGACGTGTTTAATATTCA<br>TCGGGAAGATCTTGGTATCAAGAGCGCTTTTAGCGGCGCCGCCCGGCATCTCGCTTTCGGATCCGGCATT<br>CATAACTGTGTAGGAGCAGCTTTTGCCAAAAACGAAATCGAAATTGTAGCTAATATTGTGCTGGATAAGA<br>TGCGGAATATCAGATTAGAGGAAGATTTTTGTTATGCTGAGTCCGGTCTGTATACACGCGGACCTGTTTC<br>ACTTCTCGTTGCGTTTGACGGGGCA
**CYP152A**	>lcl|Query_7269323:c152310-151060<br>EDGE_1897728_length_207403_cov_97.496613':EDGE_1889790_length_147_cov_193.800000<br>ATGAATGAGCAGATTCCACATGACAAAAGTCTCGATAACAGCCTGACACTGCTGAAGGAAGGGTATTTAT<br>TTATTAAAAACAGAACAGAGCGCTACAATTCAGATCTGTTTCAGGCCCGTTTGTTGGGAAAAAACTTTAT<br>TTGCATGACTGGCGCTGAGGCGGCGAAGGTGTTTTATGATACGGATCGATTCCAGCGGCAGAACGCTTTG<br>CCTAAGCGGGTGCAGAAATCGCTGTTTGGTGTTAATGCGATTCAGGGAATGGATGGCAGCGCGCATATCC<br>ATCGGAAGATGCTTTTTCTATCATTAATGACACCGCCGCATCAAAAACGTTTGGCTGAGTTGATGACAGA<br>GGAGTGGAGAGCAGCAGTCACAAGATGGGAGAAGACAGATGAGGTTGTGTTATTTGAAGAAGCAAAAGAA<br>ATCCTGTGCCGGGTAGCGTGCTATTGGGCAGGTGTTCCGTTGAAGGAAACGGAAGTCAAAGAGAGGGCGG<br>ATGACTTCATTGACATGGTCGACGCGTTCGGTGCTGTGGGACCGCGGCATTGGAAGGGAAGAAGAGCAAG<br>GCCGCGTGCGGAAGAGTGGATTGAAGTCATGATTGAAGATGCTCGTGCCGGCTTGCTGAAAACGACTCCC<br>GGAACAGCGCTGCATGAAATGGCTTTTCACACACAAGAAGATGGAAGCCAGCTGGATTCCCGCATGGCAG<br>CCATTGAGCTGATTAATGTACTGCGGCCTATTGTCGCCATTTCTTACTTTCTGGTGTTTTCAGCTTTGGC<br>GCTTCATGAGCATCCGAAGTATAAGGAATGGCTGCGGTCTGGAAACAGCCGGGAAAGAGAAATGTTTGTG<br>CAGGAGGTCCGCAGATATTATCCGTTCGGCCCGTTTTTAGGGGCGCTTGTCAAAAAAGATTTTGTATGGA<br>ATAACTGTGAGTTTAAAAAGGGCACATCGGTGCTGCTTGATGTATATGGAACGAACCACGACACTCGTCT<br>ATGGGAACACCCCGATGAATTCCGGCCGGAACGATTTGCGGAGCGGGAAGAAAATCCGTATGATATGATT<br>CCTCAAGGCGGGGGGCACGCCGAGAAAGGCCACCGCTGTCCAGGGGAAGGCATTACAATTGAAGTCATGA<br>AAGCGAGCCTGGATTTCCTCGTCCATCAGATTGAATACGATGTTCCGGAACAATCACTGCATTACAGTCT<br>CGCCAGAATGCCGTCATTGCCTGAAAGCGGCTTCGTAATGAGCGGAATCAGACGAAAAAGT
**CYP197A**	>lcl|Query_5483295:c660155-658818<br>EDGE_1897866_length_773419_cov_48.068913:EDGE_1872936_length_276_cov_104.161074<br>CCCAAAACATACGGACCTTTAAAAAATCTTCCGCATCTGGAAAAAGAACAGCTTTCTCAATCCTTATGGC<br>GGATAGCTGATGAATTGGGACCGATTTTCCGTTTTGATTTTCCGGGAGTATCCAGTGTTTTTGTCTCGGG<br>CCACAATTTTGTGGCTGAAGTATGTGATGAAAGCCGCTTTGACAAGAACCTTGGCAAAGGCTTGCAAAAG<br>GTGCGTGAGTTCGGAGGAGATGGCTTATTTACAAGCTGGACGCACGAACCGAACTGGCAAAAAGCCCACC<br>GCATTTTGCTGCCGAGTTTTAGTCAAAAAGCGATGAAAGGCTATCATTCTATGATGCTGGATATCGCAAC<br>CCAGCTGATTCAAAAATGGAGCCGGTTAAACCCTAATGAAGAAATTGATGTAGCGGACGATATGACACGT<br>CTGACGCTTGATACGATTGGGTTATGCGGGTTTAACTATCGATTCAACAGCTTTTACCGTGATTCACAGC<br>ATCCGTTTATCACCAGTATGCTCCGTGCCTTAAAAGAGGCGATGAATCAATCGAAAAGACTGGGCCTGCA<br>AGATAAAATGATGGTGAAAACGAAGCTGCAGTTCCAAAAGGATATAGAAGTCATGAACTCCCTGGTCGAT<br>AGAATGATAGCGGAGCGAAAGGCGAATCCGGATGAAAACATCAAGGATCTCTTGTCTCTCATGCTTTATG<br>CCAAAGATCCAGTAACCGGTGAAACGCTGGATGATGAAAACATAAGGTATCAAATCATCACATTTTTAAT<br>TGCTGGACATGAGACAACAAGCGGGTTGCTTTCCTTTGCGATTTATTGTCTGCTTACACATCCGGAAAAA<br>CTGAAAAAAGCTCAGGAGGAAGCGGATTGCGTGTTAACGGATGACACGCCTGAATATAAACAAATCCAGC<br>AGCTCAAATACATTCGGATGGTTTTAAATGAAACCCTCAGACTGTATCCAACAGCTCCGGCTTTTTCTCT<br>ATATGCGAAGGAGGATACTGTTCTAGGCGGGGAATATCCGATCAGCAAAGGGCAGCCAGTCACTGTTTTA<br>ATTCCAAAACTGCACCGGGATCAAAACGCTTGGGGACCGGATGCGGAAGATTTCCGTCCGGAACGGTTTG<br>AGGATCCTTCAAGTATCCCTCACCATGCGTATAAGCCGTTTGGAAATGGGCAGCGCGCTTGTATTGGCAT<br>GCAGTTTGCTCTTCAAGAAGCGACAATGGTTCTCGGTCTTGTATTAAAGCATTTTGAATTGATAAACCAT<br>ACTGGCTACGAACTAAAAATCAAAGAAGCATTAACGATCAAGCCGGATGATTTTAAAATTACTGTGAAAC<br>CGCGGAAA
